# The diversity and evolution of pollination systems in large plant clades: Apocynaceae as a case study

**DOI:** 10.1093/aob/mcy127

**Published:** 2018-08-07

**Authors:** Jeff Ollerton, Sigrid Liede-Schumann, Mary E Endress, Ulrich Meve, André Rodrigo Rech, Adam Shuttleworth, Héctor A Keller, Mark Fishbein, Leonardo O Alvarado-Cárdenas, Felipe W Amorim, Peter Bernhardt, Ferhat Celep, Yolanda Chirango, Fidel Chiriboga-Arroyo, Laure Civeyrel, Andrea Cocucci, Louise Cranmer, Inara Carolina da Silva-Batista, Linde de Jager, Mariana Scaramussa Deprá, Arthur Domingos-Melo, Courtney Dvorsky, Kayna Agostini, Leandro Freitas, Maria Cristina Gaglianone, Leo Galetto, Mike Gilbert, Ixchel González-Ramírez, Pablo Gorostiague, David Goyder, Leandro Hachuy-Filho, Annemarie Heiduk, Aaron Howard, Gretchen Ionta, Sofia C Islas-Hernández, Steven D Johnson, Lize Joubert, Christopher N Kaiser-Bunbury, Susan Kephart, Aroonrat Kidyoo, Suzanne Koptur, Cristiana Koschnitzke, Ellen Lamborn, Tatyana Livshultz, Isabel Cristina Machado, Salvador Marino, Lumi Mema, Ko Mochizuki, Leonor Patrícia Cerdeira Morellato, Chediel K Mrisha, Evalyne W Muiruri, Naoyuki Nakahama, Viviany Teixeira Nascimento, Clive Nuttman, Paulo Eugenio Oliveira, Craig I Peter, Sachin Punekar, Nicole Rafferty, Alessandro Rapini, Zong-Xin Ren, Claudia I Rodríguez-Flores, Liliana Rosero, Shoko Sakai, Marlies Sazima, Sandy-Lynn Steenhuisen, Ching-Wen Tan, Carolina Torres, Kristian Trøjelsgaard, Atushi Ushimaru, Milene Faria Vieira, Ana Pía Wiemer, Tadashi Yamashiro, Tarcila Nadia, Joel Queiroz, Zelma Quirino

**Affiliations:** 1 Faculty of Arts, Science and Technology, University of Northampton, Northampton, UK; 2 Lehrstuhl für Pflanzensystematik, Universität Bayreuth, Bayreuth, Germany; 3 Department of Systematic and Evolutionary Botany, University of Zurich, Zurich, Switzerland; 4 Universidade Federal dos Vales do Jequitinhonha e Mucuri (UFVJM), Curso de Licenciatura em Educação do Campo - LEC, Campus JK - Diamantina, Minas Gerais, Brazil; 5 School of Life Sciences, University of KwaZulu-Natal, Scottsville, Pietermaritzburg, South Africa; 6 Instituto de Botánica del Nordeste, UNNE-CONICET, Corrientes, Argentina; 7 Department of Plant Biology, Ecology, and Evolution, Stillwater, OK, USA; 8 Laboratorio de Plantas Vasculares, Departamento de Biología Comparada, Facultad de Ciencias, UNAM, Mexico; 9 Laboratório de Ecologia da Polinização e Interações – LEPI, Departamento de Botânica, Instituto de Biociências, Universidade Estadual Paulista “Júlio de Mesquita Filho”- Unesp, Botucatu - SP, Brazil; 10 Saint Louis University, Department of Biology, St. Louis, MO, USA; 11 Mehmet Akif Ersoy Mah. 269. Cad. Urankent Prestij Konutları, Demetevler, Ankara, Turkey; 12 Department of Biological Sciences, University of Cape Town, Rondebosch, Cape Town, South Africa; 13 Ecosystem Management Group, ETH Zurich, Switzerland; 14 EDB, UMR 5174, Université de Toulouse, UPS, Toulouse cedex, France; 15 Laboratorio de Ecología Evolutiva - Biología Floral, IMBIV (UNC-CONICET), Argentina; 16 Departamento de Botânica, Museu Nacional, Universidade Federal do Rio de Janeiro, Quinta da Boa Vista, Rio de Janiero, RJ, Brazil; 17 Department of Plant Sciences, Faculty of Natural and Agricultural Sciences, University of the Free State, Bloemfontein, South Africa; 18 Laboratório de Ciências Ambientais, Centro de Biociências e Biotecnologia, Universidade Estadual do Norte Fluminense Darcy Ribeiro, Campos dos Goytacazes-RJ, Brazil; 19 Departamento de Botânica - CB, Laboratório de Biologia Floral e Reprodutiva - POLINIZAR, Universidade Federal de Pernambuco, Recife - PE, Brazil; 20 Universidade Federal de São Carlos - UFSCar, Centro de Ciências Agrárias, Depto. Ciências da Natureza, Matemática e Educação, Araras, SP, Brazil; 21 Jardim Botânico do Rio de Janeiro, Rio de Janeiro - RJ, Brazil; 22 Facultad de Ciencias Exactas, Fisicas y Naturales, Universidad Nacional de Córdoba (UNC) and IMBIV (CONICET-UNC). CP, Córdoba, Argentina; 23 Herbarium - Royal Botanic Gardens, Kew, Richmond, Surrey, UK; 24 Laboratorio de Investigaciones Botánicas (LABIBO), Facultad de Ciencias Naturales, Universidad Nacional de Salta-CONICET. Salta, Argentina; 25 Department of Biosciences, University of Salzburg, Salzburg, Austria; 26 Biology Department, Franklin and Marshall College, Lancaster, PA, USA; 27 Natural History Museum, Georgia College, Milledgeville, GA, USA; 28 Centre for Ecology and Conservation, University of Exeter, Penryn Campus, Cornwall, UK; 29 Department of Biology, Willamette University Salem, OR, USA; 30 Department of Botany, Faculty of Science, Chulalongkorn University, Pathumwan, Bangkok, Thailand; 31 Department of Biodiversity Earth and Environmental Sciences and Academy of Natural Sciences, Drexel University, Philadephia, PA, USA; 32 Center for Ecological Research, Kyoto University, Hirano, Otsu, Shiga, Japan; 33 Universidade Estadual Paulista UNESP, Instituto de Biociências, Departamento de Botânica, Laboratório de Fenologia, Rio Claro, SP, Brazil; 34 Tanzania Wildlife Research Institute (TAWIRI), Arusha, Tanzania; 35 School of Biological Sciences, Royal Holloway University of London, Egham, Surrey, UK; 36 Graduate School of Arts and Sciences, The University of Tokyo, Komaba, Meguro-ku, Tokyo, Japan; 37 Universidade do Estado da Bahia - Campus IX. Rodovia BR, Barreiras, BA, Brazil; 38 Tropical Biology Association, Cambridge, UK; 39 Instituto de Biologia - UFU, Campus Umuarama Bloco, Uberlândia-MG, Brazil; 40 Department of Botany, Rhodes University, Grahamstown, South Africa; 41 Biospheres, Eshwari, Nanasaheb Peshva Marg, Near Ramna Ganpati, Lakshminagar, Parvati, Pune, Maharashtra, India; 42 Department of Evolution, Ecology, and Organismal Biology, University of California, Riverside, CA, USA; 43 Departamento de Biologia, Universidade Estadual de Feira de Santana, Novo Horizonte, Feira de Santana, Bahia, Brazil; 44 Key Laboratory for Plant Diversity and Biogeography of East Asia, Kunming Institute of Botany, Chinese Academy of Sciences, Kunming, Yunnan, PR China; 45 Laboratorio de Ecología, UBIPRO, FES-Iztacala, Universidad Nacional Autónoma de México, Tlalnepantla de Baz, Estado de México, México; 46 Escuela de Ciencias Biológicas, Universidad Pedagógica y Tecnológica de Colombia, Tunja, Colombia; 47 Departamento de Biologia Vegetal, Instituto de Biologia, Caixa, Universidade Estadual de Campinas, Campinas, São Paulo, Brazil; 48 Department of Plant Sciences, Natural and Agricultural Sciences, University of the Free State, Qwaqwa campus, Phuthaditjhaba, Republic of South Africa; 49 Penn State University, University Park, PA, USA; 50 Department of Chemistry and Bioscience, Aalborg University, Fredrik Bajers Vej, Aalborg, Denmark; 51 Graduate School of Human Development and Environment, Kobe University, Tsurukabuto, Kobe City, Japan; 52 Departamento de Biologia Vegetal, Universidade Federal de Viçosa (UFV), Viçosa, Minas Gerais, Brazil; 53 Museo Botánico Córdoba y Cátedra de Morfología Vegetal (IMBIV-UNC-CONICET), Córdoba, Argentina; 54 Graduate School of Technology, Industrial and Social Science, Tokushima University, Minamijyosanjima, Tokushima, Japan; 55 Centro Acadêmico de Vitória, Universidade Federal de Pernambuco, Recife, Pernambuco, Brazil; 56 Departamento de Educação, Universidade Federal da Paraiba, Mamnguape, Paraiba, Brazil; 57 Departamento de Engenharia e Meio Ambiente, Universidade Federal da Paraiba, Rio Tinto, Paraíba, Brazil

**Keywords:** Apocynaceae, Asclepiadaceae, bimodal pollination system, biogeography, fly pollination, generalization, mutualism, phylogeny, plant–pollinator interactions, pollination ecology, specialization, stapeliads

## Abstract

**Background and Aims:**

Large clades of angiosperms are often characterized by diverse interactions with pollinators, but how these pollination systems are structured phylogenetically and biogeographically is still uncertain for most families. Apocynaceae is a clade of >5300 species with a worldwide distribution. A database representing >10 % of species in the family was used to explore the diversity of pollinators and evolutionary shifts in pollination systems across major clades and regions.

**Methods:**

The database was compiled from published and unpublished reports. Plants were categorized into broad pollination systems and then subdivided to include bimodal systems. These were mapped against the five major divisions of the family, and against the smaller clades. Finally, pollination systems were mapped onto a phylogenetic reconstruction that included those species for which sequence data are available, and transition rates between pollination systems were calculated.

**Key Results:**

Most Apocynaceae are insect pollinated with few records of bird pollination. Almost three-quarters of species are pollinated by a single higher taxon (e.g. flies or moths); 7 % have bimodal pollination systems, whilst the remaining approx. 20 % are insect generalists. The less phenotypically specialized flowers of the Rauvolfioids are pollinated by a more restricted set of pollinators than are more complex flowers within the Apocynoids + Periplocoideae + Secamonoideae + Asclepiadoideae (APSA) clade. Certain combinations of bimodal pollination systems are more common than others. Some pollination systems are missing from particular regions, whilst others are over-represented.

**Conclusions:**

Within Apocynaceae, interactions with pollinators are highly structured both phylogenetically and biogeographically. Variation in transition rates between pollination systems suggest constraints on their evolution, whereas regional differences point to environmental effects such as filtering of certain pollinators from habitats. This is the most extensive analysis of its type so far attempted and gives important insights into the diversity and evolution of pollination systems in large clades.

## INTRODUCTION

Interactions between plants and their pollinators are considered to have played a major role in the diversification of some large angiosperm groups (Darwin, 1877; Crepet, 1984; Johnson, 2006; Kay and Sargent, 2009; Vamosi and Vamosi, 2010; van der Niet and Johnson, 2012; van der Niet *et al.*, 2014). Evolutionary models of reproductive isolation and adaptation to novel pollinators seem to explain species diversity in some small to modest-sized clades (e.g. Smith *et al.*, 2006; Wilson *et al.*, 2006; Whittall and Hodges, 2007; Ogutcen *et al.*, 2017 – but see Armbruster and Muchhala, 2009, for a different perspective). In other cases, such as the family Asteraceae, an evolutionary trend from specialist- to generalist-pollination systems within a clade has been suggested (Torres and Galetto, 2002). Nevertheless, most large flowering plant clades lack extensive data on pollination systems; therefore, there is limited understanding of the evolutionary transitions between different types of pollinators and the biogeographical patterns of those interactions with pollinators in large families of flowering plants. However, Apocynaceae, one of the 10–12 largest angiosperm families (species counts for families vary according to source), is geographically widespread, has a densely sampled molecular phylogeny, and has abundant field data on pollinators, representing an excellent group to address such topics.

Apocynaceae consists of at least 5350 recognized species in 378 genera (Endress *et al.*, in press). Species are distributed from tropical to temperate environments in every major biome except arctic tundra, and the family is particularly species rich in the dry and wet tropics (e.g. Li *et al*., 1995*a*, *b*; Rapini *et al.*, 2002; Rapini, 2004; Juárez-Jaimes *et al.*, 2007; Villaseñor, 2016; Ulloa Ulloa *et al.*, 2017). Growth forms in Apocynaceae cover almost the whole spectrum of plant types, including vines, scramblers, shrubs, herbs with fibrous and tuberous roots, caudiciforms, epiphytes, large and small stem succulents, leaf succulents, and small and large trees, although truly aquatic species are conspicuously absent (Ollerton, 1986; Judd *et al.*, 2002; Fishbein *et al.*, 2018).

Flowers within the family show different levels of floral synorganization and fusion of androecium and gynoecium, which has allowed the appearance of specialized pollination mechanisms, involving pollinaria, in different lineages. The highly derived pollination mechanisms of some subfamilies, particularly the Asclepiadoideae (formerly within the family Asclepiadaceae), have been studied for over two centuries (e.g. Sprengel, 1793; Brown, 1810; Delpino, 1867; Weale, 1871; Darwin, 1877; Corry, 1883; Robertson, 1886; Scott-Elliot, 1891). Moreover, two groups of Apocynaceae (Rauvolfioids and Apocynoids – see Materials and Methods) have multiple species-rich lineages with less derived flowers and simpler pollination mechanisms than those of the ‘asclepiads’ (Fallen, 1986). This permits comparative studies to elucidate the performance consequences (in terms of pollen dispersal and receipt) of derived floral morphologies (Livshultz *et al.*, 2018) and reconstruction of flower evolution that provides some a priori hypotheses for pollinator relationships (Fishbein *et al.*, 2018).

The pollination ecology of Apocynaceae is highly diverse, and there have been significant recent advances in our understanding of the pollination ecology of some major groups and across more of its global distribution (Supplementary Data S1). However, to date there has been no attempt to quantitatively synthesize what is currently known about the family as a whole. In this study we have assembled a large dataset of floral visitors and pollinators for the family, and used this to address the following questions: How much do we currently know about the diversity of pollination systems in the family? How is that diversity partitioned between the major clades of the family, and what are the evolutionary transitions between the major groups of pollinators? Do these pollination systems vary biogeographically?

Answering these questions will provide important insight into the diversity and evolution of pollination systems in a large clade of flowering plants, establish the ground work for more detailed future studies within the family, and provide a baseline for understanding pollination diversification in other major clades of angiosperms.

## MATERIALS AND METHODS

Published studies of pollinators and pollination of Apocynaceae were located by using keyword searches (Apocynac* or Asclepiad* and Pollinat*) of the major scientific depositories (e.g. Web of Science), building on the earlier literature searches of Meve and Liede (1994) and Ollerton and Liede (1997). In addition, we used our network of contacts to locate observations published in regional journals that are not always easy to obtain (e.g. Nakahama *et al.*, 2013) and to locate data in reports, theses and dissertations, as well as data held by some of the authors of this study but so far unpublished. Some of the unpublished data came from targeted fieldwork on particular groups of Apocynaceae from un(der)-studied parts of the world and from citizen science projects (see Supplementary Data S1).

### Phylogenetic and taxonomic considerations

The five major taxonomic divisions of Apocynaceae recognized here follow the most recent classifications; former subfamilies Rauvolfioideae and Apocynoideae have repeatedly been shown to be paraphyletic (Livshultz *et al.*, 2007; Straub *et al.*, 2014; Fishbein *et al.*, 2018) and are here recognized informally as Rauvolfioids and Apocynoids, respectively, following Simões *et al.* (2016), Morales *et al.* (2017) and Fishbein *et al.* (2018). Apocynoids + Periplocoideae + Secamonoideae + Asclepiadoideae (known as the APSA clade – Livshultz *et al.*, 2007) is monophyletic, and apart from a few exceptions, shares a number of reproductive morphological features that demarcates the group from Rauvolfioids. Circumscription of the major divisions as well as tribes and subtribes is mainly based on a number of molecular-based phylogenetic reconstructions (see Supplementary Data S1).

### Database construction

Data on flower visitors and pollinators of species of Apocynaceae were brought together into a single database that included details of the taxonomic placement of the species (subfamily or major division, tribe, and subtribe, as appropriate) following Endress *et al.* (in press). Plant names were updated as required and noted in the database (Supplementary Data S2).

Flower visitors were accorded a code (based on Ollerton and Liede, 1997) depending upon the quality of the data on their effectiveness as pollinators, as follows: 0 – the plant is an obligate selfer (very uncommon in Apocynaceae); 1 – identity of the pollinator proven – visitors with pollinia/pollen attached and observed to bring about pollination of a flower under natural conditions; 2 – identity of the pollinator inferred – visitors observed with pollinia/pollen attached, under natural conditions; 3 – identity of the pollinator inferred from circumstantial evidence, e.g. visitors observed on flowers, but evidence of picking up pollinia/pollen is missing, under natural conditions; 4 – the flower visitor is a nectar or pollen thief, a herbivore, a predator, or a parasite of insects in the flowers. Where pollination or visitation was observed outside of the plant’s natural range, the letter A was appended to the number code (e.g. 2A). Where pollination or visitation was observed outside of the animal’s natural range, the letter B was appended to the number code (e.g. 2B). In the database the code ‘3(2)’ indicates that although the data do not quite reach the standards of evidence required to assign them to code 2, additional evidence (e.g. details of floral phenotype) strongly supports the case for the visitors being pollinators. These were treated as code 2 in the analyses.

Details of the higher taxonomy (e.g. order, family) of the flower visitors were included, as well as the locality of the study (country) and a reference. This database will be made freely available and will be regularly updated as new information becomes available. It will supersede the APOPOL (http://132.180.63.26/planta2/research/pollina/APO_POL_d.html) and ASCLEPOL (http://132.180.63.26/planta2/research/pollina/as_pol_d.html) databases, which presently document 223 and 1562 interactions with flower visitors, respectively (Ollerton and Liede, 1997).

**Fig. 1.**
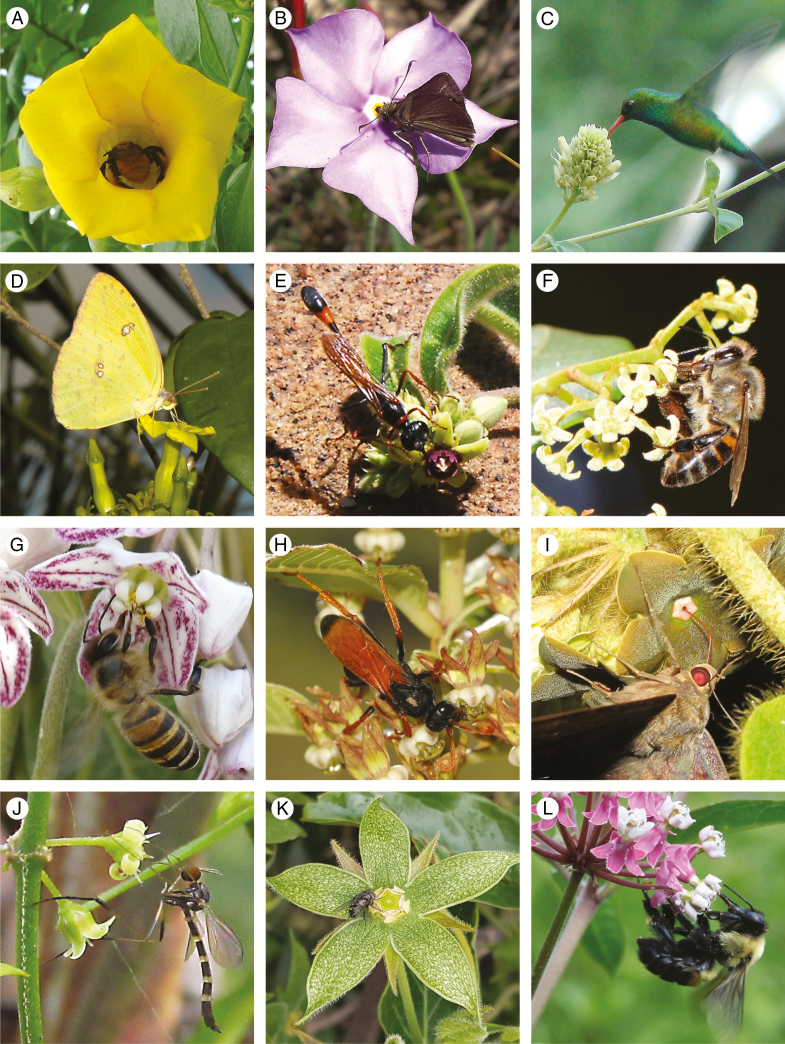
Floral visitors to Apocynaceae. (A) *Cascabela ovata* (Alvarado-Cárdenas et al . 2017) (Rauvolfioids: Plumerieae) being visited by *Eulaema* sp. (Hymenoptera: Apidae), Mexico (Photo: L. O. Alvarado-Cárdenas). (B) *Mandevilla tenuifolia* (Apocynoids: Mesechiteae) being visited by Hesperiidae sp. (Lepidoptera), Brazil (Photo: F. W. Amorim). (C) *Mandevilla pentlandiana* (Apocynoids: Mesechiteae) being visited by *Chlorostilbon lucidus* (Aves: Trochilidae), Argentina (Photo: L. Galetto). (D) *Prestonia coalita* (Apocynoids: Echiteae) being visited by *Phoebis argante* (Lepidoptera: Pieridae), Brazil (Photo: A. Rapini). (E) *Raphionacme procumbens* (Periplocoideae) being visited by *Ammophila* sp. (Hymenoptera: Sphecidae), South Africa (Photo: L. Joubert). (F) *Secamone alpini* (Secamonoideae) being visited by *Apis mellifera capensis* (Hymenoptera: Apidae), South Africa (Photo: A. Shuttleworth). (G) *Dregea sinensis* (Asclepiadoideae: Marsdenieae) being visited by *Apis cerana* (Hymenoptera: Apidae), China (Photo: Z-X. Ren). (H) *Xysmalobium orbiculare* (Asclepiadoideae: Asclepiadeae) being visited by *Hemipepsis capensis* (Hymenoptera: Pompilidae), South Africa (Photo: A. Shuttleworth). (I) *Macroscepis elliptica* (Asclepiadoideae: Asclepiadeae) being visited by *Ascalapha odorata* (Lepidoptera: Noctuidae), Argentina (Photo: H. Keller). (J) *Orthosia virgata* (Asclepiadoideae: Asclepiadeae) being visited by *Lygistorrhina edwardsi* (Diptera: Lygistorrhinidae), Argentina (Photo: H. Keller). (K) *Gonolobus grandiflorus* (Asclepiadoideae: Asclepiadeae) being visited by Sarcophagidae sp. (Diptera), Mexico (Photo: L. O. Alvarado-Cárdenas). (L) *Asclepias incarnata* (Asclepiadoideae: Asclepiadeae) being visited by *Bombus griseocollis* (Hymenoptera: Apidae), USA (Photo: N. Rafferty).

Pollinators were initially grouped into seven single taxon categories: [bee, wasp, butterfly, moth (hawkmoth + settling moth), fly, beetle, bird] plus an insect generalist category (see below). These categories were then used in our assessments of the diversity of pollinators within the family and across biogeographical regions, and for mapping pollination systems onto the phylogeny (see Figs 3, 5 and 6). For other analyses (see Fig. 4) species of Apocynaceae for which good data/evidence was available were then categorized into broad unimodal (bee, fly, wasp, bird, etc.), bimodal (e.g. bee + butterfly) and multimodal pollination systems (i.e. species pollinated by more than two broad groups of animals, e.g. bee + moth + wasp). In several of those cases (Figs 4–6), we split moth into hawkmoth and settling moth, referred to just as moth, considering the relevance and evolutionary distinctiveness of selection for hawkmoth and moth pollination. Species categorized as having a multimodal pollination system were considered to be insect generalists, although we acknowledge that this distinction between bimodal and multimodal is arbitrary to some degree. Because vertebrate pollination is rare in the family we chose to distinguish bird + insect generalist as a distinct category. A representative selection of interactions between Apocynaceae flowers and flower visitors is shown in Fig. 1.

### Phylogenetic reconstruction and mapping of pollination systems

Maximum likelihood reconstruction of ancestral states and estimation of evolutionary rates among states were conducted with the rayDISC function in the corHmm package (Beaulieu *et al.*, 2013) for R (R Core Team, 2017), following Fishbein *et al.* (2018). The root state was treated as equally likely for all characters. Three classes of models were fitted: all rates equal (ER), transition rates varying across all combinations of states that were equal forward and backward (SYM), and transition rates varying across all combinations of states that differed forward and backward (ARD). The best fitting model for each character was selected by likelihood ratio tests, and the set of adequately fitting models was found by comparing corrected Akaike Information Criterion (AICc) scores. Ancestral state reconstructions were depicted on the Apocynaceae phylogeny using the plot.phylo function in the ape v. 4.1 package (Paradis *et al.*, 2004) for R (R Core Team, 2017). Two data sets were analysed, a ‘full’ data set of 237 species, which included species where the identity of pollination systems was suspected, but not confirmed; and a ‘reduced’ data set of 135 species, for which the most confident information about pollinator type (code 1 or 2 as described above – see Supplementary Data S2B) was available. We note that the calculated transition rates may only be accurate if diversification rates are not affected by the pollination state. However, we currently do not have sufficient data to fully test this and it is a question that must be revisited in future analyses.

The base phylogeny was a chronogram (branches scaled by time) estimated from 21 concatenated plastid loci for 1041 species (Fishbein *et al.*, 2018), from which all species lacking pollination data were pruned using the drop.tip function in ape. Both the full and the reduced data sets were analysed also on a base phylogeny in which relationships along the backbone were constrained by a phylogeny of 76 complete Apocynaceae plastomes. Details of the data and analysis of these two phylogenies, as well as the differences between them, can be found in Fishbein *et al.* (2018).

Here we focus on analyses based on the plastome-constrained tree, which is more congruent with most of the recently estimated Apocynaceae phylogenies (Livshultz *et al.*, 2007; Straub *et al.*, 2014), and we present the alternative reconstructions in Supplementary Data S6.

### Data visualization

Data plots were made either using the package ‘ggplot2’ (Wickham, 2009) in R (R Core Team, 2017) or Microsoft Excel. Mapping the species richness of Apocynaceae and the number of species in the database with pollinator data was done using ArcGIS (ESRI, 2011).

## RESULTS

### Quantity and quality of available data, and the diversity of pollinators within Apocynaceae

The Pollinators of Apocynaceae Database currently contains 5061 observed interactions between pollinators and species of Apocynaceae, mainly within their natural areas, but also on some species that have been cultivated or naturalized outside of their native range (Supplementary Data S2A, 3A). From these data, 567 species can be categorized into broad pollination systems which correspond to a >10 % sample of the family (~5350 species), with representatives from all the major groups and most of the tribes and larger subtribes, although sampling is sparse or non-existent in some lineages (Supplementary Data S3C). Particularly well represented are some subtribes of Asclepiadeae and Ceropegieae (Asclepiadoideae), and the Rauvolfioid tribes Plumerieae, Aspidospermateae and Carisseae (Supplementary Data S3B, 3C).

**Fig. 2.**
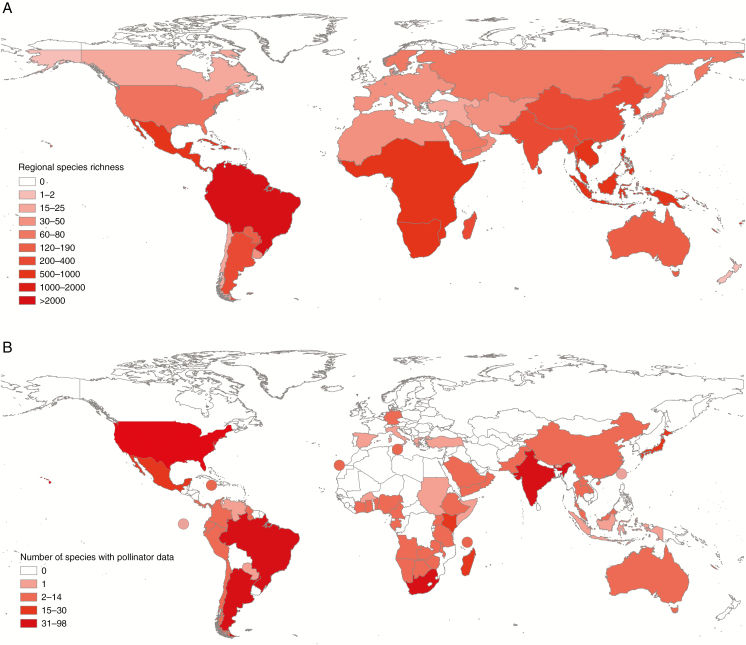
(A) Species richness of Apocynaceae mapped at a country and regional level according to available data and specialist estimates. Exact species counts are not available for most countries and the ranges used are approximations. Note that the scale used is discontinuous. (B) Geographical representation of Apocynaceae in the Pollinators of Apocynaceae Database. The colours of the countries reflect the number of species in the database with pollinator data (see key). Circles represent data from islands.

The geographical distribution of the data is both widespread and patchy with some countries being very well represented and others less so. In part this reflects the high diversity of Apocynaceae in those countries, but not completely, as some species-rich regions are not represented in the Database (Fig. 2).

The 567 species of Apocynaceae were divided into two categories: those to which we can firmly attribute a pollination system and those where we suspect (but cannot confirm) the pollination system (Supplementary Data S3A). The following analyses have been performed using only the more restricted dataset of firm attributions, comprising 294 species.

The majority (73 %) of species observed so far are pollinated by a single broad taxonomic group of animal pollinators, including bees and wasps (Hymenoptera), butterflies and moths (Lepidoptera), flies (Diptera), beetles (Coleoptera) or birds (Aves). However, there are often multiple families, genera or species involved (see the *Specialization and generalization* section below). Of the remainder, 19 % are insect generalists pollinated by at least three different major groups of pollinators (with a wide diversity of animals involved in these systems, including, in addition to the expected bees, butterflies, etc., groups such as ants and Hemiptera (Ollerton *et al.*, 2003; Domingos-Melo *et al.*, 2017). A further 7 % are bimodal, pollinated by two distinct groups of animals (Supplementary Data S5); only one species is known to be an obligate selfer [*Vincetoxicum* (*Tylophora*) *matsumurae* – see Yamashiro and Maki, 2005] although other species within this clade can autogamously self-pollinate (Liede-Schumann *et al.*, 2016).

### Evolutionary transitions of plant-pollinator interactions

**Fig. 3.**
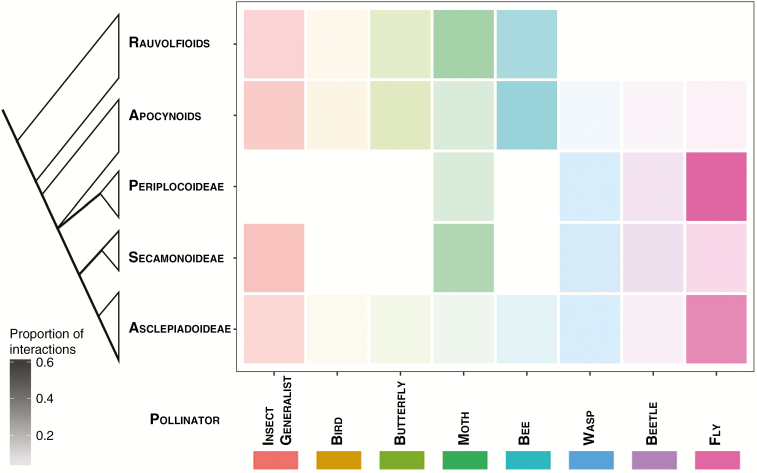
Phylogenetic relationships among the major groups of Apocynaceae with their known pollinators. Colour intensities reflect the proportion of plant species within each major group that is pollinated by a given type of pollinator. Note that only confirmed pollinators have been mapped against this phylogeny with the exception of Secamonoideae where the sparsity of observations means that suspected (but not confirmed) pollinators have been mapped (Supplementary Data S3).

**Fig. 4.**
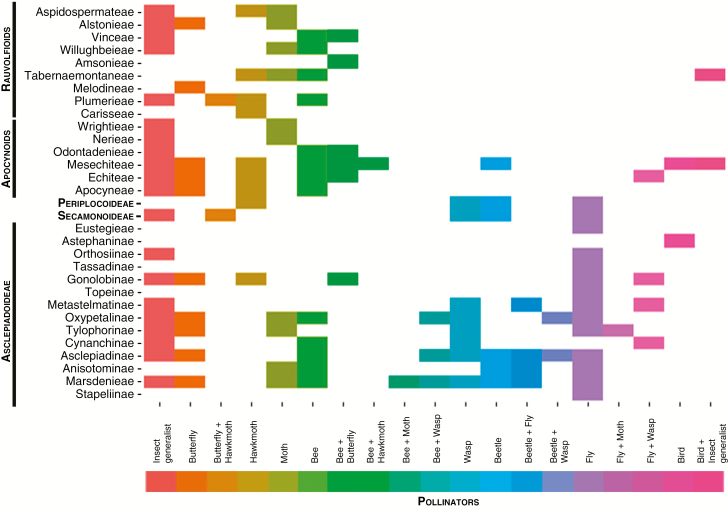
Pollination systems within major divisions, tribes and subtribes of Apocynaceae. Only confirmed pollinators have been mapped against this phylogeny with the exception of Secamonoideae where the sparsity of observations means that suspected (but not confirmed) pollinators have been mapped (Supplementary Data S3A and 3B). Pollination systems have been categorized into those with only a single major group of pollinators and those with two (‘bimodal’). Tribes and subtribes follow Endress *et al.* (2014) and are roughly ordered evolutionarily from less (top) to more (bottom) derived.

At a broad systematic and pollination system scale there is a clear phylogenetic structure within the Apocynaceae regarding which pollinator types are used by members of the different taxa and clades (Fig. 3). Species along the earliest diverging grade formed by the tribes of Rauvolfioids exploit a rather restricted set of pollinators compared with the APSA clade. Beetle and wasp pollination are restricted to the more derived tribes of Apocynoids and the subfamilies Periplocoideae, Secamonoideae and Asclepiadoideae (Fig. 4). The use of a broad range of insects (‘insect generalist’) as well as bees, moths and butterflies as pollinators is widely distributed across the family.

Fly pollination, one of the distinctive features of members of the subtribe Stapeliinae (Ceropegieae) and subtribe Gonolobineae (Asclepiadeae), is actually widespread throughout the Periplocoideae and Asclepiadoideae, and also found in some derived Apocynoids (although only together with wasps) (Fig. 4, Supplementary Data S3).

Birds, particularly sunbirds (Nectariniidae) and hummingbirds (Trochilidae) are frequent flower visitors to Apocynaceae but the degree to which they rob nectar from otherwise insect-pollinated flowers is unclear. If the birds recorded as visitors to flowers in the early diverging groups are legitimate pollinators then bird pollination may have arisen several times, often bimodally with insect generalist pollination. Within Asclepiadoideae bird pollination has been confirmed from Astephaninae where pollinia transfer occurs on birds’ tongues (Pauw, 1998). Whether this can also occur with free pollen from Rauvolfioids or Apocynoids remains to be determined.

### Reconstructing the evolution of pollination systems

Of the 294 species to which we can firmly attribute pollination systems (with code 1 and 2 pollinator observations), 135 are represented in the plastid phylogeny. The best fitting model for the evolution of this reduced data set analysed on the plastome-constrained phylogeny selected by the hierarchical likelihood-ratio test was the symmetric (SYM) model, though the equal-rates model (ER) was selected based on AICc (Supplementary Data S6, Table S1). Because strong heterogeneity in transition rates is evident (Table S5), we focus interpretation on the SYM model. Under this model (Table S5), only nine of the 28 possible pollination transitions are inferred to have non-zero rates. The highest transition rates are estimated for switches between wasp and beetle pollination; this rate is >100× greater than any other transition. The second most frequent transition (at least 5× greater than the remaining) occurs between hawkmoth and settling moth pollination. All pollination types have non-zero transition rates to at least two other categories, although some systems are more constrained. Transitions away from beetle pollination almost always occur to wasps, and the reverse is almost as pronounced. The next most restricted pollination types are butterfly, which has a low rate of transition only to bee or moth, and fly pollination, which has a low rate of transition to only hawkmoth or general insect pollination. These patterns are largely consistent with those found with the full dataset of 238 species with less stringent criteria for attributing pollinators (Table S3).

**Fig. 5.**
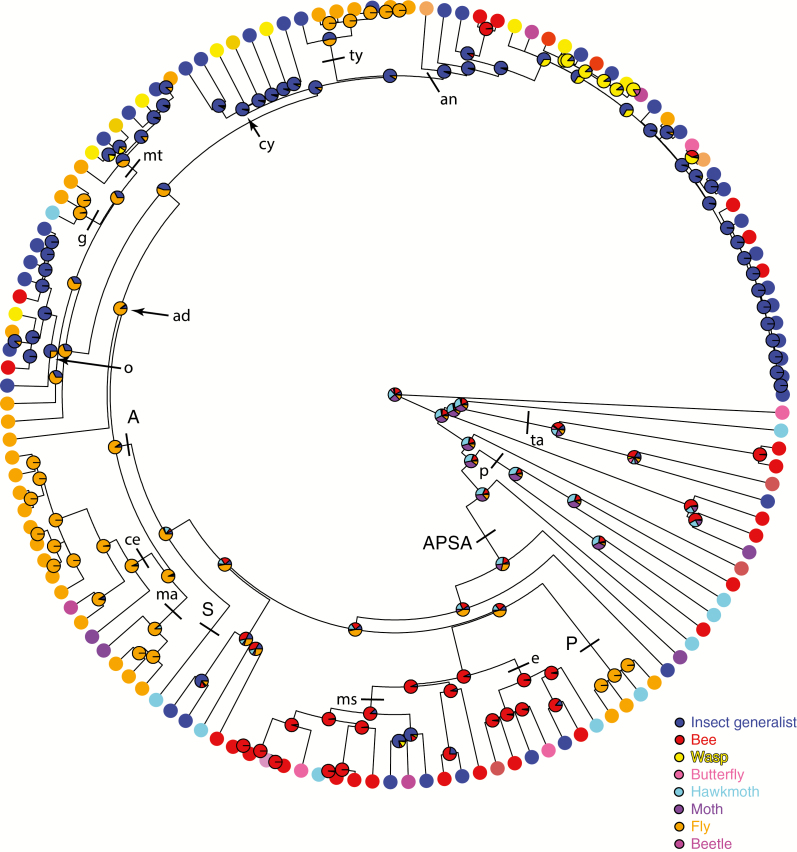
Pollinator types mapped onto a phylogeny of Apocynaceae. Maximum likelihood estimates of ancestral states of pollinator type for the reduced data set depicted on the chronogram in Supplementary Data S6 (Fig. S2). Pollinator types are indicated as in the key with polymorphic states indicated by additional intermediate shades of colour. Probabilities of states at ancestral nodes are indicated by pie charts. Best-fitting evolutionary models and rates are given in Supplementary Data S6 (Tables S1, S5). Major clades are indicated by tick marks or arrows and labelled as follows: Apocynoids–Periplocoideae–Secamonoideae–Asclepiadoideae (APSA); subfamilies: Periplocoideae (P), Secamonoideae (S) and Asclepiadoideae (A); tribes: Asclepiadeae (ad), Ceropegieae (ce), Echiteae (e), Marsdenieae (ma), Mesechiteae (ms), Plumerieae (p) and Tabernaemontaneae (ta); and subtribes: Asclepiadinae (an), Cynanchinae (cy), Gonolobinae (g), Metastelmatinae (mt), Oxypetalinae (o) and Tylophorinae (ty).

Across the Apocynaceae, pollination systems have been regularly lost and gained over time (Fig. 5, Supplementary Data S6). There is great lability in pollinator associations within most major grades/clades. Shifts early in the diversification of the family reduce certainty in reconstructing ancestral pollinators throughout the Rauvolfioid grade. This is also especially apparent for the large APSA clade, whose ancestor is reconstructed as equally likely to have been pollinated by hawkmoths or flies, and nearly as likely to have been pollinated by settling moths or bees. Bee pollination is inferred to be the ancestral state for the common ancestor of Mesechiteae, Odontadenieae and Echiteae (Apocynoid grade). Asclepiadoideae are inferred to be ancestrally fly-pollinated, which is retained in the common ancestor of Asclepiadeae, followed by a major shift to general insect pollination in the common ancestor of Cynanchinae, Tylophorinae and Asclepiadinae. There is an independent shift to general insect pollination inferred for Oxypetalinae. The only major clade with constrained pollinator associations is Marsdenieae–Ceropegieae, in which ancestral fly pollination is retained in most extant species (Fig. 5, Supplementary Data S6).

These results are, however, quite sensitive to sampling and data quality. Analysis of the full data set (including species with tentative, unconfirmed assignments of pollination systems) shows retention of fly pollination in Asclepiadoideae further into the diversification of the subtribes, with Oxypetalinae and the Cynanchinae–Tylophorinae–Asclepiadinae clade having greater probabilities of being ancestrally fly-pollinated (Supplementary Data S6). There is also more ambiguity as to whether Mesechiteae–Odontadenieae–Echiteae were ancestrally bee- or general insect-pollinated. Although these reconstructions are supported by increased sampling, this comes at the cost of including less reliable data. Increased sampling also suggests that the ancestral pollinators of Secamonoideae were hawkmoths, those of Periplocoideae were flies and those of Tabernaemontaneae were butterflies or settling moths. There is also greater probability that pollinators during the early diversification of the family were bees (Supplementary Data S6).

#### Biogeographical patterns of plant–pollinator interactions

**Fig. 6.**
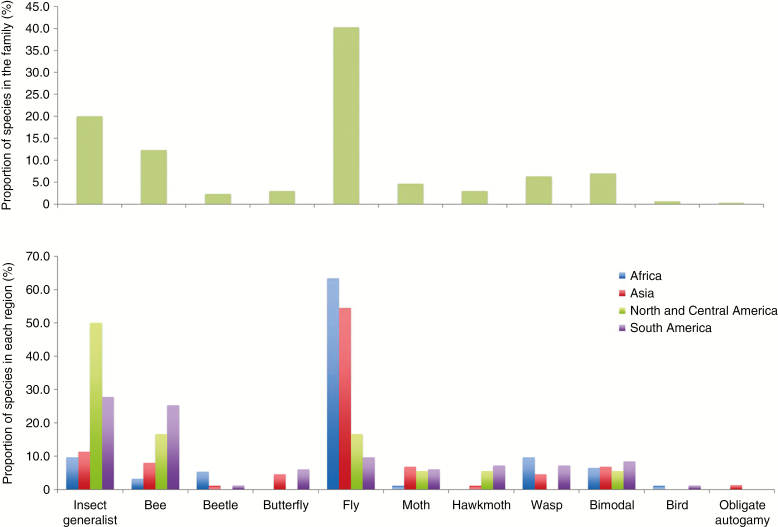
Proportion of species of Apocynaceae per pollination system (above), and their geographical representativeness (below). Only those regions with large samples of Apocynaceae species are included in the comparison.

Our data allow broad comparisons of plant–pollinator interactions for species in four regions: Asia, Africa, North and Central America, and South America (Fig. 6, Supplementary Data S4). Compared to the spectrum of pollinators recorded for the family as a whole, some striking patterns are apparent. Fly pollination is much more frequent in Africa and Asia in comparison with the Americas, although this may be affected by the large amount of recent work on *Ceropegia* and its relatives (see Ollerton *et al.*, 2017 for a summary) as the large subtribe Gonolobinae, restricted to the Americas, is also mainly fly-pollinated (see below). In the Americas, bee and insect generalist pollination are more common compared to the other regions (Fig. 6) but it is notable that, in general, specialized pollination by bees is not as common as one might expect given the dominance of these insects as pollinators of other plant groups (Ollerton, 2017).

Specialized butterfly pollination from Africa, and beetle and wasp pollination from North and Central America, has not yet been reported, but is suspected but not confirmed for some species (see Supplementary Data S3 and S4).

**Fig. 7.**
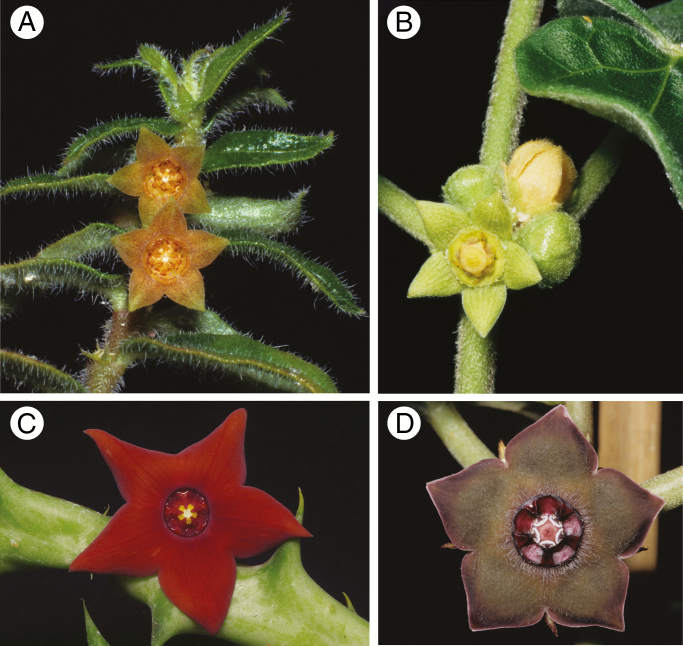
Flowers showing characteristic traits of fly pollination in Ceropegieae (A, C, left column) and Asclepiadeae–Gonolobinae (B, D, right column). (A) *Brachystelma* (*Ceropegia*) *simplex*, Ivory Coast. (B) *Ibatia ganglinosa*, Brazil. (C) *Orbea sprengeri* subsp. *commutata*, Saudi Arabia. (D) *Matelea cyclophylla*, Mexico. (Photos: U. Meve).

There are some striking patterns of convergent evolution between distantly related, biogeographically separated groups. For example, fly pollination in Stapeliinae and Gonolobinae has resulted in the evolution of similar flower colours, patterns, textures and odours (Fig. 7). However, fly-trap pollination of the type found in *Ceropegia* and *Riocreuxia*, and very large, fleshy *Stapelia*-like ‘carrion flowers’ are restricted to the Old World, and absent from the New World Gonolobinae. Similarly, moth pollination shows convergent evolution between clades and regions, as for example in species of *Schubertia* (Asclepiadoideae: Gonolobinae) and *Aspidosperma* (Rauvolfioids: Aspidospermateae) in South America, *Dictyophleba lucida* (Rauvolfioids: Willughbeieae) in Africa and *Telosma cordata* (Asclepiadoideae: Marsdenieae) in India.

Levels of specialization also tend to vary between regions, and South African ecosystems are particularly well known for high levels of specialization (Johnson and Steiner, 2000, 2003), including the subfamily Asclepiadoideae (Ollerton *et al.*, 2006). Pollination systems in South African Asclepiadoideae typically involve a single functional type of pollinator, and include several unusual pollination systems. Specialized pollination by *Hemipepsis* spider-hunting wasps (Pompilidae: Pepsinae), for example, appears to be unique to South African ecosystems and mainly involves asclepiads (it is currently known to occur in 17 species from seven asclepiad genera; Shuttleworth and Johnson, 2012). Although functionally similar spider-hunting wasps visit or pollinate asclepiads in other geographical regions (Punzo, 2006; Wiemer *et al.*, 2012), they represent components of much broader assemblages of pollinators and do not represent the sole pollinators of these species as they do in the South African systems.

Chafer beetles (Scarabaeidae: Cetoniinae) are another particularly important group of pollinators in South African grassland ecosystems (Peter and Johnson, 2009, 2013; Steenhuisen and Johnson, 2012), and represent specialist pollinators for some asclepiads (Ollerton *et al.*, 2003; Shuttleworth and Johnson, 2009*a*). Specialized pollination by chafer beetles has been confirmed in seven species from four genera, but is likely to be considerably more frequent in the region. Chafer-pollinated asclepiads in South Africa are mostly reliant on the beetle *Atrichelaphinis tigrina* but *Cyrtothyrea marginalis* is also often involved and one species, *Pachycarpus scaber*, appears to be specialized to this second beetle (Ollerton *et al.*, 2003; Shuttleworth and Johnson, 2009*a*). Finally, pollination by sunbirds has been established in the red-flowered South African *Microloma sagittatum* (Pauw, 1998), and represents the only known example of bird pollination within the subfamily Asclepiadoideae. Bird pollination is particularly unusual in this instance as it involves the attachment of pollinaria to the birds’ tongues. The *Microloma* flowers involved also exhibit strong convergence with other bird-pollinated flowers (red colouring and a tubular corolla; Ollerton, 1998).

It is also interesting to note that in southern Africa (Asclepiadinae) and South America (Oxypetalinae) there have been parallel shifts between wasp (Vespidae and Pompilidae) and beetle pollination, particularly to flower chafers (Cetoniinae).

### Specialization and generalization in Apocynaceae

Almost three-quarters of the species have unimodal pollination systems involving a single major group of insects, or birds alone (Supplementary Data S5). However, within these functionally specialized (*sensu*Ollerton *et al.*, 2007) pollination systems, multiple species, genera or even families of insects are frequently involved, making them ecologically more generalized. Pollination by a single species is extremely rare in the family and its detection is limited by methodological biases because the number of pollinators observed for a species generally increases with sampling effort (i.e. hours of observation and number of populations observed – see Ollerton *et al.*, 2003 and Supplementary Data S1).

Fewer than 10 % of the species for which we have data seem to have bimodal pollination systems involving two distinct groups of animals. Although the sample size is limited, some combinations of pollinators are more common than others, for example bee + butterfly and beetle + fly, whilst other combinations have not yet been recorded (Table 1).

**Table 1.** The number of Apocynaceae species engaged in bimodal pollination relationships with two distinct groups of pollinators

	Bee	Beetle	Butterfly	Fly	Moth + Hawkmoth	Wasp	Insect generalist
Beetle	0						
Butterfly	7	0					
Fly	0	1	0				
Moth + Hawkmoth	2	0	1	1			
Wasp	3	6	0	2	0		
Bird	0	0	0	0	0	0	2

The most specialized Apocynaceae studied to date are some *Ceropegia* spp. and related stapeliads, where a single genus or species of Diptera may be the sole pollinator (Ollerton *et al.*, 2009; Heiduk *et al*., 2010, 2015, 2016, 2017), and some of the South African asclepiads from the grasslands which are also typically pollinated by a single species or genus of pompilid wasp or cetoniid beetle (Ollerton *et al.*, 2003; Shuttleworth and Johnson, 2009*a*, *b*, *c*; see the *Biogeographical patterns* section above).

## DISCUSSION

The evolutionary and biogeographical patterns of plant–pollinator interactions evidenced in Apocynaceae show a complex interplay of constraints and flexibility that we are just beginning to appreciate. Apocynaceae exploit pollen vectors from most of the main animal groups known to act as pollinators (as recently summarized by Ollerton, 2017) with the exception of lizards and mammals, and, with some rare exceptions, birds. In addition, pollination by wind and water is unknown, and obligate selfing extremely rare. Mapping these pollination systems onto the phylogeny of species within the Pollinators of Apocynaceae Database, and subsequent ancestral state reconstruction (Fig. 5), shows that certain clades are associated with a rather conservative range of pollinators, e.g. fly pollination in Stapeliinae. Other clades are conservative with respect to the broad range of pollinators that individual species use, e.g. insect generalist *Asclepias* species in North America (although this may be biased by over-representation of the common, widespread species that are more likely to be generalists). However, there are also groups such as Mesechiteae where evolutionary flexibility and frequent switches between pollination systems has occurred.

The highest rate of transition on the phylogeny between pollination systems is between wasp and beetle pollination, which is more than 100 times that of any of the other transitions. This suggests that flowers pollinated by wasps and beetles are similar in their floral phenotype and the resources they offer. This is supported by the high number plants with wasp + beetle bimodal pollination (Table 1). However, the most frequent bimodal pollination system is bee + butterfly, but the rate of shifts between these pollinators is not high. In addition, Table 1 suggests to us that there may be some constraints on which bimodal interactions can evolve, perhaps due to limitations of particular sensory modalities or nectar rewards, for example presence of amino acids or specific ratios of sugars. One could view this as analogous to Stebbins’ finding that certain combinations of characters occur repeatedly in different lineages, whereas other combinations are never found together, phenomena which he referred to as adaptive peaks and valleys (Stebbins, 1950). It would thus be interesting to disentangle what drivers and constraints determine how bimodal interactions can evolve within the different clades of Apocynaceae, considering that they have frequently evolved during the diversification of this plant family. Deeper understanding of these patterns, and the processes underlying them, will require additional detailed field data on pollinators from some of the more species-rich groups. In addition, we need a better appreciation of the relationships between the floral morphologies in these clades and the diversity of pollinators, and whether there are some morphological traits that facilitate diversification and others which prevent it.

One particularly striking finding is that in the APSA clade, with more derived floral phenotypes, pollination by anthophilous insects (those that depend on and are highly adapted to floral resources such as bees and butterflies) is much less frequent than in the Rauvolfioids. The APSA clade contains many species that are pollinated by flies, wasps and beetles which are often less dependent on flowers to complete their life cycles and often lack traits such as long proboscides, or pollen- or oil-collecting structures. This has been a successful strategy for clades such as Asclepiadoideae and one explanation may be that, by exploiting groups of pollinators that are less frequently used by other species, they can open up new adaptive pollination niches in which there is less competition for pollinators (see also Ollerton *et al.*, 2003). It is possible that the evolution of highly aggregated and efficient pollen transfer mechanisms with pollinia and translators was a key innovation that permitted exploitation of these less behaviourally optimized pollinators (Livshultz *et al*., 2011, 2018).

There is a pattern of adding pollen vectors as flower complexity in terms of synorganization increases (Fig. 3). The elaborate five-part ‘revolver’ flowers and the diverse gynostegial coronas are features that could favour the selection and canalizing of different types of pollinators (Endress, 1996, 2015; Fishbein, 2001). However, in groups such as *Asclepias*, Cynanchinae and Oxypetalinae it has not precluded the evolution of highly generalized interactions. Generalist pollination in more derived clades has also been suggested for other groups, including *Dalechampia* (Armbruster and Baldwin, 1998), Asteraceae (Torres and Galetto, 2002) and *Miconia* (de Brito *et al.*, 2017). Further behavioural work is needed to determine the interactions of floral elements, such as coronas, and different types and assemblages of pollinators. Some of these aspects have been recently studied in genera such as *Mandevilla* and *Araujia* in South America (Moré *et al.*, 2007; Araújo *et al.*, 2014; Wiemer *et al.*, 2012) and in southern African groups (see above). However, the diversity of coronas in Apocynaceae and the range of physical and behavioural characteristics of pollen vectors deserves a thorough evaluation.

Another important finding from our study relates to the range of pollination systems within large monophyletic groups. Two of the largest subtribes/tribes within Apocynaceae, with 720–730 species each, are characterized by possession of one (Stapeliinae) and ten (Marsdenieae) distinct pollination systems (Fig. 4). Stapeliinae is well represented in the Pollinators of Apocynaceae Database (Supplementary Data S2 and 3A) and has diversified rapidly across Africa and Asia over the last 10 million years (Bruyns *et al.*, 2015; Fishbein *et al.*, 2018) into a species radiation that has involved only fly pollination. Previously, pollinator shifts between major groups of pollinators (e.g. bird to bee) have been suggested as a significant driver of plant diversification and termed the Grant–Stebbins model (Johnson, 2006). This has not occurred in Stapeliinae although there is evidence for it in Marsdenieae, the sister clade to Ceropegieae wherein Stapeliinae are nested. However, there is nothing in the Grant–Stebbins model to preclude what may appear to be ‘minor’ shifts of pollinators (i.e. fly to fly) from playing a role in the diversification of large clades. The biology of Diptera is hugely varied, and this is reflected in the diversity of different forms of fly pollination (Ollerton and Raguso, 2006). For example, in the genus *Ceropegia*, fly pollination can take a number of forms, including deception of kleptoparasitic Diptera (Heiduk *et al*., 2010, 2015, 2016, 2017) as well as mimicry of fermenting or rotting substrates (Ollerton *et al.*, 2009) and rewarding, generalized flowers (Coombs *et al.*, 2011). Diptera may contain several functional pollinator groups and involve distinctive floral adaptations; for example, some plants pollinated by fungus gnats (Mycetophilidae) exhibit similar floral traits (Mochizuki and Kawakita, 2017). Therefore, ‘minor’ shifts of pollinators may be just as significant as ‘major’ shifts for diversification, i.e. the pattern seen in Stapeliinae is qualitatively similar to that seen in Marsdenieae, but at a different (pollinator) phylogenetic level. There is no reason to suppose that this is confined to Diptera; it may equally apply to other groups of pollinators such as bees.

### Strengths and future applications of the Pollinators of Apocynaceae Database

The Pollinators of Apocynaceae Database is the largest and most extensive compilation of such data that has ever been assembled for a plant family of this size. It contains a >10 % sample of species within the family with data on flower visitors and pollinators (Supplementary Data S2), with a wide phylogenetic and geographical coverage. As a freely available resource, the database will in the future be used to explore many other questions, such as how evolution of complex flowers, pollinaria and rewards (or rewardlessness) has been influenced by the type of pollinators that a flower attracts and exploits. Additionally, this database will serve to guide efforts in the systematic collection of data in poorly studied parts of the world, and for incompletely known taxa of Apocynaceae. An important future value of the Pollinators of Apocynaceae Database will be to assess a number of conservation issues. These include the extent to which introduced honey-bees (*Apis mellifera*) and other pollinators are affecting plant reproduction (and potentially selection on floral traits) as well as the ability of introduced, invasive Apocynaceae to co-opt native pollinators, for example the South American *Araujia sericifera* that uses honey-bees as its pollinator in South Africa (Coombs and Peter, 2010). Plant-pollinator interactions–pollinator interactions within the family present different degrees of specialization at ecological, functional or phenotypic levels (*sensu*Ollerton *et al.*, 2007). This information could be used to inform conservation of native habitats that maintain populations of Apocynaceae, in which their pollinators can be supported by other plant species and nesting opportunities.

## CONCLUSIONS

In this study we have shown that Apocynaceae is probably one of the best-studied large families from the perspective of understanding the diversity of pollinators that interact with flowering plants. The pattern of evolution of pollination systems within Apocynaceae shows significant phylogenetic structure, with more frequent transitions between some pollinator types than others. The morphologically less derived clades are pollinated by a narrower range of pollinators, which is a surprising finding as one might expect that more complex floral morphology would restrict certain types of pollinators. There is also considerable biogeographical structure to the distribution of pollination systems; some regions lack particular interactions with pollinators that in other regions are extremely common.

It is possible that some of the patterns we are observing, especially in relation to ancestral state reconstruction and rates of transition, are due to under-sampling. However, in assessing pollinators of different groups within Apocynaceae as a whole, we have been conservative in our attribution of pollination systems to species. Inclusion of those pollination systems that we suspect are present in some clades (but cannot confirm) increases the diversity of pollination systems in most clades. For example, bird pollination appears more frequently across the family (but always in combination with insects). Otherwise this does not alter our broad conclusions for the most part. Therefore, as always, the findings from this study need to be tempered with the knowledge that there is limited sampling for some species in our analysis, and some lineages of Apocynaceae are not represented at all. Some of these clades have recently been shown to be of critical importance for understanding the evolution of complex floral characters in the family, for example the Baisseeae which is sister to the Secamonoideae + Asclepiadoideae (Livshultz *et al.*, 2007; Fishbein *et al.*, 2018).

Bat pollination has never been confirmed within the family; however, the database contains one record of unidentified Apocynaceae pollen on bats in Brazil, and we are also aware of images circulating on the internet showing bats visiting Apocynaceae flowers in Costa Rica. There are also intriguing flowers such as those of the mass-flowering *Mandevilla veraguasensis* in Central America that bear some of the hallmarks of specialized bat-pollinated flowers, being dull dusky purplish-brown, large, funnel-shaped and pendant on relatively long pedicels (M. E. Endress, pers. obs.). Therefore, the question of whether bat pollination occurs in Apocynaceae deserves further study.

The biogeographical findings from this study indicate that the ecological context in which these plants have evolved their interactions with pollinators would be an interesting area to explore in more detail in the future. This could include potential links between growth form, habitat type and pollination system, as has been proposed for the pollinia-bearing Secamonoideae plus Asclepiadoideae (Livshultz *et al.*, 2011) and documented in Araceae (Chouteau *et al.*, 2008). In addition, historical climate has been shown to affect current relationships between plants and their pollinators (Dalsgaard *et al.*, 2013). It is therefore likely that the environmental selective forces defining the plant communities in which these Apocynaceae exist have played a role in the evolution and diversification of pollination systems by excluding certain types of pollinators from those communities.

As far as we know our study is the most extensive and detailed of its kind yet attempted. However, a >10 % sample of species from such a large family as Apocynaceae, and with a highly non-random geographical distribution of data, means that there is undoubtedly still much to discover as we evaluate evolutionary pathways across this diverse clade of plants.

## SUPPLEMENTARY DATA

Supplementary data are available online at https://academic.oup.com/aob and consist of the following. S1: Additional Materials and Methods. S2A: Pollinators of Apocynaceae Database – all entries. S2B: Description of the codes used to assign quality to the entries in the Pollinators of Apocynaceae Database. S2C: References for the Pollinators of Apocynaceae Database. S3A: Assignment of the Apocynaceae species to broad pollination systems. S3B: Assignment of the pollination systems to groups within Apocynaceae. S3C: Species richness within groups of Apocynaceae and the number of species in each group with good pollinator data. S4: Biogeographical assignment of species in S2A. S5: Data on levels of specialization of species of Apocynaceae. S6: Results from phylogenetic analysis of species of Apocynaceae represented in the Pollinators of Apocynaceae Database.

Supplementary Material S1

Supplementary Material S2

Supplementary Material S3

Supplementary Material S4

Supplementary Material S5

Supplementary Material S6
