# Supplementary material for: The diversity and evolution of pollination systems in large plant clades: Apocynaceae as a case study
Source: Ann Bot. 2018 Aug 7;123(2):311–25. doi: 10.1093/aob/mcy127 (PMC6344220; doi:10.1093/aob/mcy127)
Supplement: Supplementary Material S1 [file mcy127_suppl_supplementary_material-s01.docx]

**Supplementary Information 1: additional Materials and Methods**

*Pollinator data*

Some genera of Apocynaceae have been relatively well studied, in depth if not in breadth. For example, North American *Asclepias* remains a model system for understanding ecological and evolutionary phenomena such as pollinator sharing within sympatric populations and inflorescence “design” (e.g. Kephart, 1983; Wyatt and Broyles, 1994; Stoepler *et al*., 2012). Our knowledge has improved for other genera in the last decade, for example *Ceropegia* (Masinde, 2004: Ollerton *et al*., 2009a, 2017; Heiduk *et al*., 2010, 2015, 2016, 2017), *Pachycarpus* (Ollerton *et al*., 2003; Shuttleworth and Johnson, 2006, 2009a, 2009b, 2009c), *Tylophora* and *Vincetoxicum* (Yamashiro *et al*., 2008), *Oxypetalum* (Vieira and Shepherd, 1999), and *Mandevilla* (Moré *et al*., 2007; Araújo *et al*., 2014). Other major groups of Apocynaceae are only beginning to be studied, such as Periplocoideae (e.g. Pisciotta *et al*., 2011).

However there are whole clades for which we have no published data on pollination ecology (e.g. Fockeeae, Secamonoideae) though some of us have collected (currently unpublished) data on some of these groups in previously poorly studied parts of the world. For example, from 1993 onwards the first author has undertaken field work in Australia, Gabon, Namibia, South Africa, Guyana, Venezuela, Peru, Tenerife and Tanzania which was wholly or at least in part focused on asclepiads and other Apocynaceae. Some of the results of this field work have already been published, either as accounts of pollination ecology (Ollerton and Liede, 2003; Ollerton *et al.,* 2003, 2009a) or as data points in broader syntheses (Ollerton and Cranmer, 2002; Ollerton *et al.,* 2006, 2009b, 2011). However much of the data collected by JO remain formally unpublished. Likewise data from field work in Madagascar by LC, focused on *Secamone* (a genus for which there are no published pollination studies) was included in the following analyses. Similarly LPCM contributed some unpublished data from early or recent work on seasonal forest and *campo rupestre* (rocky mountain grasslands), for genera such as such as *Gonolobus* and *Minaria*. In other areas with important Apocynaceae diversity, such as Mexico, biologists are starting to collect data about visitors and potential pollinators (and the few existing available surveys are also included here). Likewise, SP has been working on *Ceropegia* and *Brachystelma* in India and collected much unpublished data (Punekar 2015). Also, some data were focused on a specific pollination system such as López-Uribe *et al.* (2008) for bee pollination, Araújo and Sazima (2003) for birds, Amorim (2012) for hawkmoths, Somavilla and Kohler (2012) for wasps. Observations were also obtained by CIP from LepiMAP, a citizen science project documenting the distribution of Lepidoptera in Africa and hosted by the University of Cape Town’s “Virtual Museum” (http://vmus.adu.org.za/).

*Apocynaceae taxonomy and phylogeny*

Studies of Apocynaceae taxonomy and phylogeny that informed our work, especially in relation to the summary phylogeny presented in Fig. 3 in the main text, and the names and circumscription of higher taxa include: Liede and Täuber, 2000, 2002; Potgieter and Albert, 2001; Meve and Liede, 2002, 2004a, b; Sennblad and Bremer, 2002; Rapini *et al.*, 2003, 2006, 2007, 2011; Simões *et al.*, 2004, 2006, 2007, 2010, 2016; Liede-Schumann *et al.*, 2005, 2012, 2014, 2016; Endress *et al.*, 2007; Goyder *et al.*, 2007; Ionta and Judd, 2007; Livshultz, 2010; Livshultz *et al.* 2007, 2018; Krings *et al.*, 2008; Fishbein *et al.*, 2011, 2018; Hechem *et al.*, 2011; Silva *et al.*, 2011; Middleton and Livshultz, 2012; Bruyns *et al.*, 2017; Joubert *et al.*, 2016; Khanum *et al.*, 2016; Yang *et al.*, 2016; Chuba *et al.*, 2017; Meve *et al.*, 2017; Morales *et al.*, 2017).

*Spatio-temporal completeness of pollinator sampling*

The effect of additional sampling of pollinators over space and time is strikingly illustrated by the data we have for *Asclepias syriaca* in North America, one of the 19% of species that are insect generalists. Despite being studied for almost 100 years, we are still adding species to the list of its pollinators and this shows no sign of levelling off (see Fig. 1.1). This species is therefore highly generalised at a population level (alpha diversity) and shows significant turnover of pollinators between populations (beta diversity).

Many of the recently observed visitors to *Asclepias syriaca* are important pollinators and include lepidopterans, lamyprid beetles, and *Bombus* and megachilid bees. Lepidopterans are often considered to be nectar robbers of *A. syriaca*, but several studies (Morse and Fritz 1983 and Jennersten and Morse 1991) indicate that they play a meaningful part in fruit production and those described most recently contributed to a statistically significant rate of outcross pollen deposition (Howard and Barrows 2014). Lampyrid beetles have been observed visiting flowers at many different sites (Aaron Howard, pers. obs, Harmony Dalgleish, pers. comm.) and have been observed carrying pollinaria (Faust and Faust 2014). Bumble bees are very common pollinators of *A. syriaca*, and even if the abundance of specific species may vary greatly over time and by geographic location, they are generally efficient transporters of pollen (Howard and Barrows 2014). Less is known about the Megachilidae visitors, but they are known to be very common in some locations and can carry significant amounts of pollen (Betz et al. 1994 and Howard, unpublished data). Thus surveys of new populations add significantly to the known diversity of important pollinators of the species.


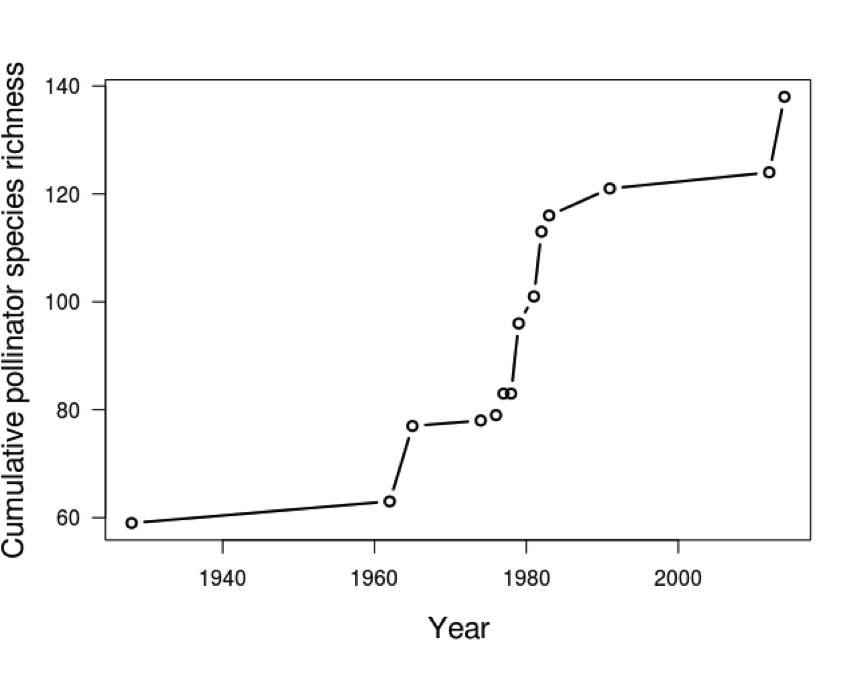


Figure 1.1: Cumulative plot of the number of recorded pollinators of *Asclepias syriaca* over time for different populations and surveys in the USA.

**LITERATURE CITED**

**Amorim FW. 2012.** *A flora esfingófila de uma floresta ombrófila densa montana no sudeste brasileiro e relações mutualísticas com a fauna de Sphingidae.* Tese (Doutorado), Universidade Estadual de Campinas, Brasil.

**Araújo AC, Sazima M. 2003.** The assemblage of flowers visited by hummingbirds in the “capões” of Southern Pantanal, Mato Grosso do Sul, Brazil. *Flora* **198**: 427-435.

**Araújo LDA, Quirino ZGM, Machado IC. 2014**. High specialisation in the pollination system of *Mandevilla tenuifolia* (J.C. Mikan) Woodson (Apocynaceae) drives the effectiveness of butterflies as pollinators. *Plant Biology* **16**: 947-955.

**Betz RF, Struven RD, Wall JE, Heitler FB. 1994.** Insect pollinators of 12 milkweed (*Asclepias*) species. In: Wickett RG, Lewis PD, Woodliffe A, Pratt P, eds. Proceedings of the Thirteenth North American Prairie Conference: Spirit of the land, our prairie legacy. Windsor, Ontario: Department of Parks & Recreation, pp 45-60.

**Bruyns PV, Klak C, Hanáček P. 2017**. A revised, phylogenetically-based concept of *Ceropegia* (Apocynaceae). *South African Journal of Botany* **112**: 399–436.

**Chuba D, Goyder DJ, Chase MW, Fishbein M. 2017.** Phylogenetics of the African *Asclepias* complex (Apocynaceae) based on three plastid DNA regions. *Systematic Botany* **42**: 148–159.

**Endress ME, van der Ham RWJM, Nilsson S, *et al*. 2007**. A phylogenetic analysis of Alyxieae (Apocynaceae) based on *rbc*L, *mat*K, *trn*L intron, *trn*L-F spacer sequences, and morphological characters. *Annals of the Missouri Botanical Garden* **94**: 1–35.

**Faust L, Faust H. 2014.** The occurrence and behaviors of North American fireflies (Coleoptera: Lampyridae) on Milkweed, *Asclepias syriaca* L. *The Coleopterists Bulletin* **68**: 283-291.

**Fishbein M, Chuba D, Ellison C, Mason-Gamer RJ, Lynch SP. 2011.** Phylogenetic relationships of *Asclepias* (Apocynaceae) estimated from non-coding cpDNA sequences. *Systematic Botany* **36**: 1008–1023.

**Fishbein M, Livshultz T, Straub SCK, Simões AO, Boutte J, McDonnell A, Foote A. 2018.** Evolution on the backbone: Apocynaceae phylogenomics and new perspectives on growth forms, flowers, and fruits. *American Journal of Botany* **105**: 495-513.

**Goyder D, Nicholas A, Liede-Schumann S. 2007.** Phylogenetic relationships in subtribe Asclepiadinae (Apocynaceae: Asclepiadoideae). *Annals of the Missouri Botanical Garden* **94**: 423–434.

**Hechem V, Calviño CI, Ezcurra C. 2011.** Molecular phylogeny of *Diplolepis* (Apocynaceae-Asclepiadoideae) and allied genera, and taxonomic implications. *Taxon* **60**: 638–648.

**Heiduk A, Brake I, Tolasch, *et al.* 2010**. Scent chemistry and pollinator attraction in the deceptive trap flowers of *Ceropegia dolichophylla*. *South African Journal of Botany* **76**: 762–769.

**Heiduk A, Kong H, Brake I, *et al.* 2015**. Deceptive *Ceropegia dolichophylla* fools its kleptoparasitic fly pollinators with exceptional floral scent. *Frontiers in Ecology and Evolution* **3**: 66.

**Heiduk A, Brake I, von Tschirnhaus M, *et al.* 2016**. *Ceropegia sandersonii* mimics attacked honeybees to attract kleptoparasitic flies for pollination. *Current Biology* **26**: 2787–2793.

**Heiduk A, Brake I, von Tschirnhaus M, *et al.* 2017**. Floral scent and pollinators of *Ceropegia* trap flowers. *Flora* **232**: 169-182.

**Howard AF, Barrows EM. 2014.** Self-pollination rate and floral-display size in *Asclepias syriaca* (Common Milkweed) with regard to floral-visitor taxa. *BMC Evolutionary Biology* **14**: 144. https://doi.org/10.1186/1471-2148-14-144.

**Ionta GM, Judd WS. 2007.** Phylogenetic relationships in Periplocoideae (Apocynaceae s.l.) and insights into the origin of pollinia in the subfamily. *Annals of the Missouri Botanical Garden* **94**: 360–375.

**Jennersten O, Morse DH. 1991.** The quality of pollination by diurnal and nocturnal insects visiting common milkweed *Asclepias syriaca. American Midland Naturalist* **125**: 18-28.

**Joubert L, Klak C, Venter AM, Venter HJT, Bruyns PV. 2016.** A widespread radiation in the Periplocoideae (Apocynaceae): The case of *Cryptolepis*. *Taxon* **65**: 487–501.

**Kephart SR. 1983**. The partitioning of pollinators among three species of *Asclepias*. *Ecology* **64**: 120-133.

**Khanum R, Surveswaran S, Meve U, Liede-Schumann S. 2016.** *Cynanchum* (Apocynaceae: Asclepiadoideae): A pantropical Asclepiadoid genus revisited. *Taxon* **65**: 467–486.

**Krings A, Thomas DT, Xiang QY. 2008.** On the generic circumscription of *Gonolobus* (Apocynaceae, Asclepiadoideae): Evidence from molecules and morphology. *Systematic Botany* **33**: 403–415.

**Liede S, Täuber A. 2000.** *Sarcostemma* R. Br. (Apocynaceae - Asclepiadoideae) - a controversial generic circumscription reconsidered: Evidence from *trn*L-F Spacers. *Plant Systematics and Evolution* **225**: 133–140.

**Liede S, Täuber A. 2002.** Circumscription of the genus *Cynanchum* (Apocynaceae - Asclepiadoideae). *Systematic Botany* **27**: 789–800.

**Liede-Schumann S, Rapini A, Goyder DJ, Chase MW. 2005.** Phylogenetics of the New World subtribes of Asclepiadeae (Apocynaceae-Asclepiadoideae): Metastelmatinae, Oxypetalinae, and Gonolobinae. *Systematic Botany* **30**: 184–200.

**Liede-Schumann S, Kong H-H, Meve U, Thiv M. 2012.** *Vincetoxicum* and *Tylophora* (Apocynaceae: Asclepiadoideae: Asclepiadeae)–two sides of the same medal: Independent shifts from tropical to temperate habitats. *Taxon* **61**: 803–825.

**Liede-Schumann S, Nikolaus M, Soares e Silva UC, Rapini A, Mangelsdorff RD, Meve U. 2014.** Phylogenetics and biogeography of the genus *Metastelma* (Apocynaceae-Asclepiadoideae-Asclepiadeae: Metastelmatinae). *Systematic Botany* **39**: 594–612.

**Liede-Schumann S, Khanum R, Mumtaz AS, Gherghel I, Pahlevani A. 2016.** Going west – A subtropical lineage (*Vincetoxicum*, Apocynaceae: Asclepiadoideae) expanding into Europe. *Molecular Phylogenetics and Evolution* **94**: 436–446.

**Livshultz T. 2010.** The phylogenetic position of milkweeds (Apocynaceae subfamilies Secamonoideae and Asclepiadoideae): Evidence from the nucleus and chloroplast. *Taxon* **59**: 1016–1030.

**Livshultz T, Middleton DJ, Endress ME, Williams JK. 2007.** Phylogeny of Apocynoideae and the APSA clade (Apocynaceae). *Annals of the Missouri Botanical Garden* **94**: 324–359.

**Livshultz T, Middleton DJ, Van der Ham RWJM, Khew G. 2018.** Generic delimitation in Apocyneae (Apocynaceae). *Taxon* **67**: 341–358.

**López-Uribe MM, Oi CA, Del Lama MA. 2008.** Nectar-foraging behavior of euglossine bees (Hymenoptera: Apidae) in urban areas. *Apidologie* **39**: 410–418.

**Masinde PS. 2004**. Trap-flower fly pollination in East African *Ceropegia* L. (Apocynaceae). *International Journal of Tropical Insect Science* **24**: 55-72.

**Meve U, Liede S. 2002.** A molecular phylogeny and generic rearrangement of the stapelioid Ceropegieae (Apocynaceae-Asclepiadoideae). *Plant Systematics and Evolution* **234**: 171–209.

**Meve U, Liede S. 2004*a***. Generic delimitations in tuberous Periplocoideae (Apocynaceae) from Africa and Madagascar. *Annals of Botany* **93**: 407–414.

**Meve U, Liede S. 2004*b***. Subtribal division of Ceropegieae (Apocynaceae-Asclepiadoideae). *Taxon* **53**: 61–72.

**Meve U, Heiduk A, Liede-Schumann S. 2017.** Origin and early evolution of Ceropegieae (Apocynaceae-Asclepiadoideae). *Systematics and Biodiversity* **15**: 143–155.

**Middleton DJ, Livshultz T. 2012.** *Streptoechites* gen. nov., a new genus of Asian Apocynaceae. *Adansonia, sér. 3,* **34**: 365–375.

**Morales J, Endress ME, Liede-Schumann S. 2017.** Sex, drugs and pupusas: Disentangling relationships in Echiteae (Apocynaceae). *Taxon* **66**: 623–644.

**Moré M, Sércic AN, Cocucci AA. 2007**. Restriction of pollinator assemblage through flower length and width in three long-tongued hawkmoth-pollinated species of *Mandevilla* (Apocynaceae, Apocynoideae). *Annals of the Missouri Botanical Garden* **94**: 485-504.

**Morse DH, Fritz RS. 1983.** Contributions of diurnal and nocturnal insects to the pollination of common milkweed (*Asclepias syriaca* L.) in a pollen-limited system. *Oecologia* 60: 190-197.

**Ollerton J, Dötterl S, Ghorpadé K, *et al*. 2017**. Diversity of Diptera families that pollinate *Ceropegia* (Apocynaceae) trap flowers: an update in light of new data and phylogenetic analyses. *Flora* **234**: 233-244.

**Ollerton J, Cranmer L. 2002**. Latitudinal trends in plant-pollinator interactions: are tropical plants more specialised? *Oikos* **98**: 340-350.

**Ollerton J, Liede S. 2003**. Corona structure in *Cynanchum*: linking morphology to function. *Ecotropica* **9**: 107-112.

**Ollerton J, Johnson SD, Cranmer L, Kellie S. 2003**. The pollination ecology of an assemblage of grassland asclepiads in South Africa. *Annals of Botany* **92**: 807-834.

**Ollerton J, Johnson SD, Hingston AB. 2006**. Geographical variation in diversity and specificity of pollination systems. In: Waser NM, Ollerton J, eds. *Plant-Pollinator Interactions: from Specialization to Generalization*. Chicago: University of Chicago Press, 283-308.

**Ollerton J, Masinde S, Meve U, Picker M, Whittington A. 2009a**. Fly pollination in *Ceropegia* (Apocynaceae: Asclepiadoideae): Biogeographic and phylogenetic perspectives. *Annals of Botany* **103**: 1501-1514.

**Ollerton J, Alarcón R, Waser NM, *et al*. 2009b**. A global test of the pollination syndrome hypothesis. *Annals of Botany* **103**: 1471-1480.

**Ollerton J, Tarrant S, Winfree R. 2011**. How many flowering plants are pollinated by animals? *Oikos* **120**: 321-326.

**Pisciotta S, Raspi A, Sajeva M. 2011**. First records of pollinators of two co-occurring Mediterranean Apocynaceae. *Plant Biosystems* **145**: 141-149.

**Potgieter K, Albert VA.** **2001.** Phylognetic relationhips within Apocynaceae s.l. bsed on *trnL* intron and *trnL-F* spacer sequences and propagule characters*. Annals of the Missouri Botanical Garden* **88**: 523–549.

**Punekar SA. 2015.** Molecular systematics, phylogeny and ecology of *Ceropegia* L. (Apocynaceae-Asclepiadoideae) in India. Report submitted to Science and Engineering Research Board, Department of Science and Technology.

**Rapini A, Chase MW, Goyder DJ, Griffiths J. 2003.** Asclepiadeae classification: evaluating the phylogenetic relationships of New World Asclepiadoideae (Apocynaceae). *Taxon* **52**: 33–50.

**Rapini A, Chase MW, Konno, TUP. 2006.** Phylogenetics of South American Asclepiadeae (Apocynaceae). *Taxon* **55:** 119-124.

**Rapini A, van den Berg C, Liede-Schumann L. 2007.** Diversification of Asclepiadoideae (Apocynaceae) in the New World. *Annals of the Missouri Botanical Garden* **94**: 407–422.

**Rapini A, Fontella Pereira J, Goyder DJ. 2011.** Towards a stable generic circumscription in Oxypetalinae (Apocynaceae). *Phytotaxa* **26**: 9–16.

**Sennblad B, Bremer B. 2002.** Classification of Apocynaceae s.l. according to a new approach combining Linnaean and phylognentic taxonomy. *Systematic Biology* **5**: 389–409.

**Shuttleworth A, Johnson SD. 2006**. Specialized pollination by large spider-hunting wasps and self-incompatibility in the African milkweed *Pachycarpus asperifolius*. *International Journal of Plant Sciences* **167**: 1177-1186.

**Shuttleworth A, Johnson SD. 2009a**. Palp-faction: an African milkweed dismembers its wasp pollinators. *Environmental Entomology* **38**: 741-747.

**Shuttleworth A, Johnson SD. 2009b.** The importance of scent and nectar filters in a specialized wasp-pollination system. *Functional Ecology* **23:** 931-940.

**Shuttleworth A, Johnson SD. 2009c.** New records of insect pollinators for South African asclepiads (Apocynaceae: Asclepiadoideae). *South African Journal of Botany* **75:** 689-698.

**Silva UCS, Rapini A, Liede-Schumann S, Ribeiro, PL, van den Berg C. 2011.** Taxonomic considerations on Metastelmatinae (Apocynaceae) based on plastid and nuclear DNA. *Systematic Botany* **37**: 795-806.

**Simões AO, Endress ME, van der Niet T, Conti E, Kinoshita LS. 2004.** Tribal and intergeneric relationships of Mesechiteae (Apocynoideae, Apocynaceae): evidence from three noncoding plastid DNA regions and morphology. *American Journal of Botany* **91**: 1409–1418.

**Simões AO, Endress ME, van der Niet T, Kinoshita LS, Conti E. 2006.** Is *Mandevilla* (Apocynaceae, Mesechiteae) monophyletic? Evidence from five plastid DNA loci and morphology. *Annals of the Missouri Botanical Garden* **93**: 565–591.

**Simões AO, Livshultz T, Conti E, Endress ME. 2007.** Phylogeny and systematics of the Rauvolfioideae (Apocynaceae) based on molecular and morphological evidence. *Annals of the Missouri Botanical Garden* **94**: 268–297.

**Simões AO, Endress ME, Conti E. 2010.** Systematics and character evolution of Tabernaemontaneae (Apocynaceae, Rauvolfioideae) based on molecular and morphological evidence. *Taxon* **59**: 772–790.

**Simões AO, Kinoshita LS,** **Koch I, Silva MJ, Endress ME. 2016.** Systematics and character evolution of Vinceae (Apocynaceae). *Taxon* **65**: 99–122.

**Somavilla A, Köhler A. 2012.** Preferência floral de vespas (Hymenoptera, Vespidae) no Rio Grande do Sul, Brasil. *Entomobrasilis* 5: 21-28.

**Stoepler TM, Edge A, Steel R, O'Quinn L, Fishbein M. 2012.** Differential pollinator effectiveness and importance in a milkweed (*Asclepias*, Apocynaceae) hybrid zone. *American Journal of Botany* **99**: 448-458.

**Vieira MF, Shepherd GJ. 1999**. Pollinators of *Oxypetalum* (Asclepiadaceae) in southeastern Brazil. *Revista Brasileira de Biologia* **59**: 693-704.

**Wyatt R, Broyles SB. 1994**. Ecology and evolution of reproduction in milkweeds. *Annual Review of Ecology and Systematics* **25**: 423-441.

**Yamashiro T, Yamashiro A, Yokoyama J, Maki M. 2008**. Morphological aspects and phylogenetic analyses of pollination systems in the *Tylophora*-*Vincetoxicum* complex (Apocynaceae-Asclepiadoideae) in Japan. *Biological Journal of the Linnean Society* **93**: 325-341.

**Yang, LL, Li HL, Wei L, *et al.* 2016**. A supermatrix approach provides a comprehensive genus‐level phylogeny for Gentianales. *Journal of Systematics and Evolution* **54**: 400-415.
