# Supplementary material for: The diversity and evolution of pollination systems in large plant clades: Apocynaceae as a case study
Source: Ann Bot. 2018 Aug 7;123(2):311–25. doi: 10.1093/aob/mcy127 (PMC6344220; doi:10.1093/aob/mcy127)
Supplement: Supplementary Material S3 [file mcy127_suppl_supplementary_material-s03.docx]

**Supplementary Information 6 – Tables and figures for phylogenetic reconstruction**

Suppl. Table 1. Evolutionary model selection in a likelihood framework for pollinator type. Model selection is shown for two data sets, full and reduced (see text for details), analyzed on two phylogenies based on concatenated sequences of 21 plastid loci, unconstrained and constrained by backbone relationships estimated from a plastome dataset (see Fishbein et al., 2018).

| Character | Model | lnL | AICc | dAICc | wAICc |
| --- | --- | --- | --- | --- | --- |
| Full-Unconstrained | **ARD** | **-345.6591** | 899.4158 | 67.9886 | 0 |
|  | SYM | -373.056 | **831.7272** | **0** | **1** |
|  | ER | -425.9681 | 853.9532 | 22.5260 | 0 |
| Full-Constrained | **ARD** | **-341.1086** | 890.3147 | 62.7339 | 0 |
|  | SYM | -371.1304 | **827.5808** | **0** | **0.998** |
|  | ER | -419.1992 | 840.4154 | 12.8346 | 0.002 |
| Reduced-Unconstrained | ARD | -182.0877 | 558.0217 | 98.2197 | 0 |
|  | **SYM** | **-195.3200** | 461.9607 | 2.1587 | 0.254 |
|  | ER | -228.8860 | **459.8020** | **0** | **0.746** |
| Reduced-Constrained | ARD | -181.0448 | 555.9358 | 95.8134 | 0 |
|  | **SYM** | **-196.8959** | 465.1126 | 4.9903 | 0.0762 |
|  | ER | -229.0461 | **460.1224** | **0** | **0.9238** |

Notes: AICc= corrected Akaike Information Criterion, ARD=all rates different model, dAICc =the difference between the AICc of the model and the best model, ER=equal rates model, lnL=log-likelihood of the model, SYM=symmetric model, wAICc=AICc weight of the model. **Bold** font in the Model and lnL columns indicates the selected model by the likelihood ratio test; **bold** font in the AICc, dAICc, and wAICc columns indicates model selected by AICc comparison and any model within two AICc units.

Suppl. Table 2. Evolutionary rates for pollinator type transitions on Apocynaceae plastid phylogeny for the full dataset. Transition rates are shown from row state to column state.

|  | General | Bee | Wasp | Butterfly | Hawkmoth | Moth | Fly | Beetle | Bird |
| --- | --- | --- | --- | --- | --- | --- | --- | --- | --- |
| General |  | 0.00229 | 0.003249 | 0.000416 | 0 | 0 | 0.00144 | 0 | 0 |
| Bee | 0 |  | 0 | 0 | 0.000981 | 0.000885 | 0 | 0 | 0 |
| Wasp | 0.00501 | 0 |  | 0 | 0 | 0 | 0 | 0.166 | 0 |
| Butterfly | 0 | 0 | 0 |  | 0 | 0.00234 | 0 | 0 | 0.00153 |
| Hawkmoth | 0.00362 | 0 | 0.000202 | 0 |  | 0 | 0 | 0 | 0 |
| Moth | 0 | 0 | 0 | 0.00401 | 0 |  | 0 | 0 | 0.00286 |
| Fly | 0.00179 | 0 | 4.09 x 10^-5^ | 0 | 7.25 x 10^-5^ | 0.000166 |  | 0 | 0 |
| Beetle | 0.0118 | 0 | 0.550 | 0 | 0 | 0 | 0 |  | 0 |
| Bird | 0 | 0.00389 | 0 | 0 | 0 | 0.000907 | 0 | 0 |  |

Suppl. Table 3. Evolutionary rates for pollinator type transitions on Apocynaceae constrained phylogeny for the full dataset. Transition rates are shown from row state to column state.

|  | General | Bee | Wasp | Butterfly | Hawkmoth | Moth | Fly | Beetle | Bird |
| --- | --- | --- | --- | --- | --- | --- | --- | --- | --- |
| General |  | 0.00205 | 0 | 0 | 0 | 0 | 0.000149 | 0.00447 | 0 |
| Bee | 0 |  | 0 | 0 | 0.000803 | 0.000544 | 9.30 x 10^-5^ | 0 | 0.000219 |
| Wasp | 0.00966 | 0 |  | 0.00101 | 0 | 0 | 0.00174 | 0.157 | 0 |
| Butterfly | 0 | 0 | 0 |  | 0 | 0.00174 | 0 | 0 | 0.00262 |
| Hawkmoth | 0.00377 | 0 | 0 | 0 |  | 0 | 0 | 0 | 0 |
| Moth | 0 | 0 | 0 | 0.00497 | 0 |  | 0 | 0 | 0.00128 |
| Fly | 0.00140 | 0 | 0 | 0 | 0.000178 | 0.000193 |  | 0 | 0 |
| Beetle | 0 | 0 | 0.500 | 0 | 0 | 0 | 0 |  | 0 |
| Bird | 0 | 0.00323 | 0 | 0 | 0 | 0.000232 | 0 | 0 |  |

Suppl. Table 4. Evolutionary rates for pollinator type transitions on Apocynaceae plastid phylogeny for the reduced dataset. Transition rates are shown from row state to column state.

|  | General | Bee | Wasp | Butterfly | Hawkmoth | Moth | Fly | Beetle |
| --- | --- | --- | --- | --- | --- | --- | --- | --- |
| General |  | 0.000710 | 0.000596 | 0 | 0 | 0 | 0.000664 | 0 |
| Bee | 0.000710 |  | 0 | 0.000451 | 0.000321 | 0 | 0 | 0 |
| Wasp | 0.000596 | 0 |  | 0 | 0 | 0 | 0 | 0.337 |
| Butterfly | 0 | 0.000451 | 0 |  | 0 | 0.000489 | 0 | 0 |
| Hawkmoth | 0 | 0.000321 | 0 | 0 |  | 0.00212 | 0.000427 | 0 |
| Moth | 0 | 0 | 0 | 0.000489 | 0.00212 |  | 0 | 0 |
| Fly | 0.000664 | 0 | 0 | 0 | 0.000427 | 0 |  | 0 |
| Beetle | 0 | 0 | 0.337 | 0 | 0 | 0 | 3.38 x 10^-5^ |  |

Suppl. Table 5. Evolutionary rates for pollinator type transitions on Apocynaceae constrained phylogeny for the reduced dataset. Transition rates are shown from row state to column state.

|  | General | Bee | Wasp | Butterfly | Hawkmoth | Moth | Fly | Beetle |
| --- | --- | --- | --- | --- | --- | --- | --- | --- |
| General |  | 0.000674 | 0.00108 | 0 | 0 | 0 | 0.000652 | 0 |
| Bee | 0.000674 |  | 0 | 0.000219 | 0.000498 | 0 | 0 | 0 |
| Wasp | 0.00108 | 0 |  | 0.000264 | 0 | 0 | 0 | 0.00188 |
| Butterfly | 0 | 0.000219 | 0.000264 |  | 0 | 2.74 x 10^-5^ | 0 | 0 |
| Hawkmoth | 0 | 0.000498 | 0 | 0 |  | 0.00290 | 0.000450 | 0 |
| Moth | 0 | 0 | 0 | 2.74 x 10^-5^ | 0.00290 |  | 0 | 0 |
| Fly | 0.000652 | 0 | 0 | 0 | 0.000450 | 0 |  | 6.44 x 10^-5^ |
| Beetle | 0 | 0 | 0.00188 | 0 | 0 | 0 | 6.44 x 10^-5^ |  |

Suppl. Fig 1. Chronogram of 237 Apocynaceae species pruned from an analysis of 1041 species (Fishbein et al., 2018). The base tree was obtained from penalized-likelihood smoothing of substitution rates across a maximum likelihood phylogeny estimated from 21 plastid loci. Major clades are indicated by tick marks or arrows and labeled as follows: Apocynoids-Periplocoideae-Secamonoideae-Asclepiadoideae (APSA); subfamilies: Periplocoideae (P), Secamonoideae (S), and Asclepiadoideae (A); tribes: Asclepiadeae (ad), Ceropegieae (ce), Echiteae (e), Marsdenieae (ma), Mesechiteae (ms), Plumeriae (p), and Tabernaemontaneae (ta); and subtribes Asclepiadinae (an), Cynanchinae (cy), Gonolobinae (g), Metastelmatinae (mt), Oxypetalinae (o), and Tylophorinae (ty).

Suppl. Fig 2. Chronogram of 135 Apocynaceae species pruned from an analysis of 1041 species (Fishbein et al., 2018). The base tree was obtained from penalized-likelihood smoothing of substitution rates across a maximum likelihood phylogeny estimated from 21 plastid loci. Major clades are indicated by tick marks or arrows and labeled as follows: Apocynoids-Periplocoideae-Secamonoideae-Asclepiadoideae (APSA); subfamilies: Periplocoideae (P), Secamonoideae (S), and Asclepiadoideae (A); tribes: Asclepiadeae (ad), Ceropegieae (ce), Echiteae (e), Marsdenieae (ma), Mesechiteae (ms), Plumeriae (p), and Tabernaemontaneae (ta); and subtribes Asclepiadinae (an), Cynanchinae (cy), Gonolobinae (g), Metastelmatinae (mt), Oxypetalinae (o), and Tylophorinae (ty).

Suppl. Fig. 3. Maximum likelihood estimates of ancestral states of Apocynaceae pollinator type for the full data set depicted on the chronogram in Fig. 1. Clade abbreviations follow Fig. 1. Pollinator types are indicated as in the legend with polymorphic states indicated by additional intermediate shades of color. Probabilities of states at ancestral nodes are indicated by pie charts. Best-fitting evolutionary models and rates are in Tables 1, 3.

**LITERATURE CITED**

**Fishbein M, Livshultz T, Straub SCK, Simões AO, Boutte J, McDonnell A, Foote A. 2018.** Evolution on the backbone: Apocynaceae phylogenomics and new perspectives on growth forms, flowers, and fruits. *American Journal of Botany* **105**: 495-513.
