# Supplementary figures and images for: The diversity and evolution of pollination systems in large plant clades: Apocynaceae as a case study
Source: Ann Bot. 2018 Aug 7;123(2):311–25. doi: 10.1093/aob/mcy127 (PMC6344220; doi:10.1093/aob/mcy127)

## Slide 1
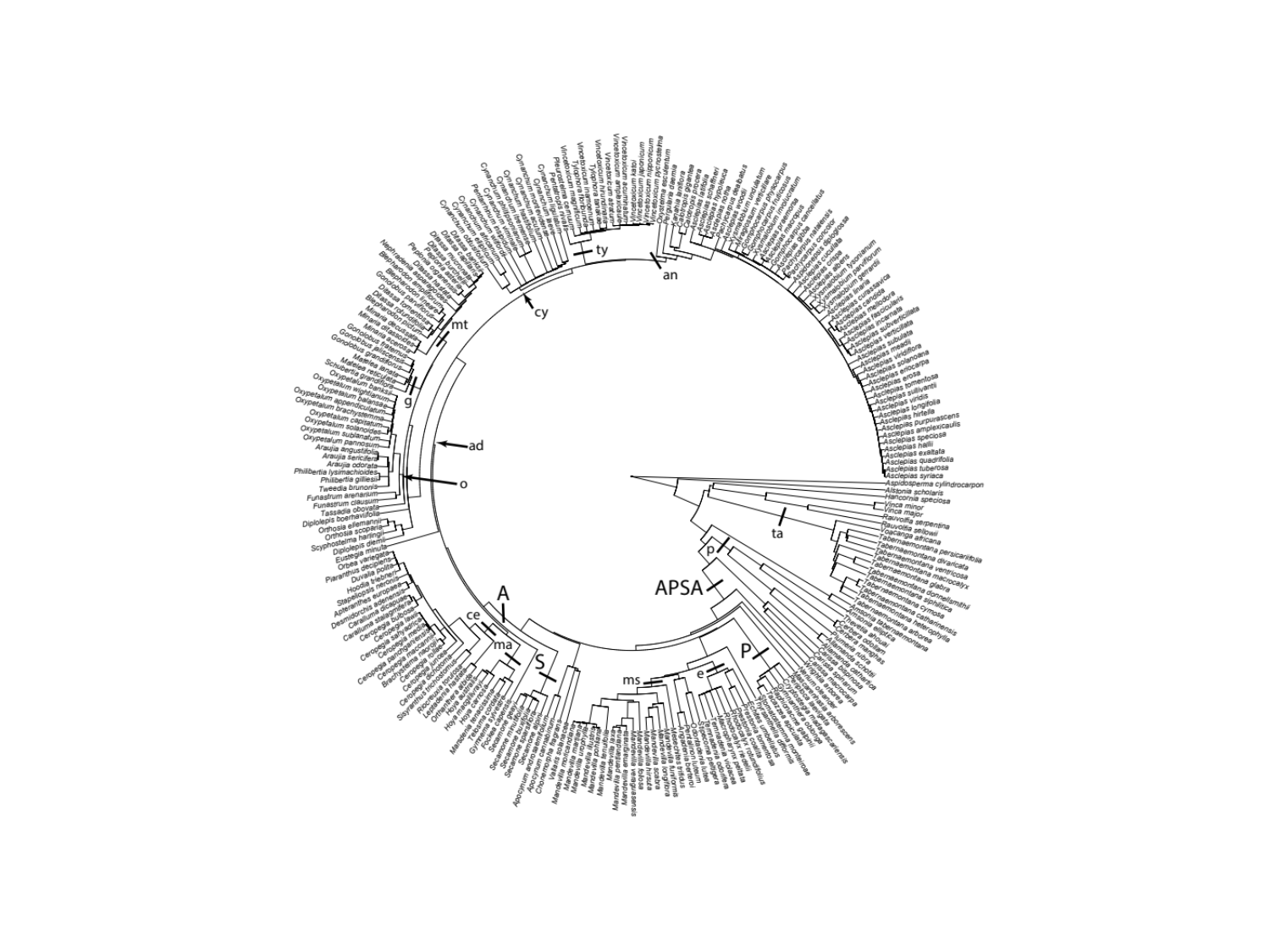

Supplement: Supplementary Material S4 [file mcy127_suppl_supplementary_material-s04.pptx]

## Slide 1
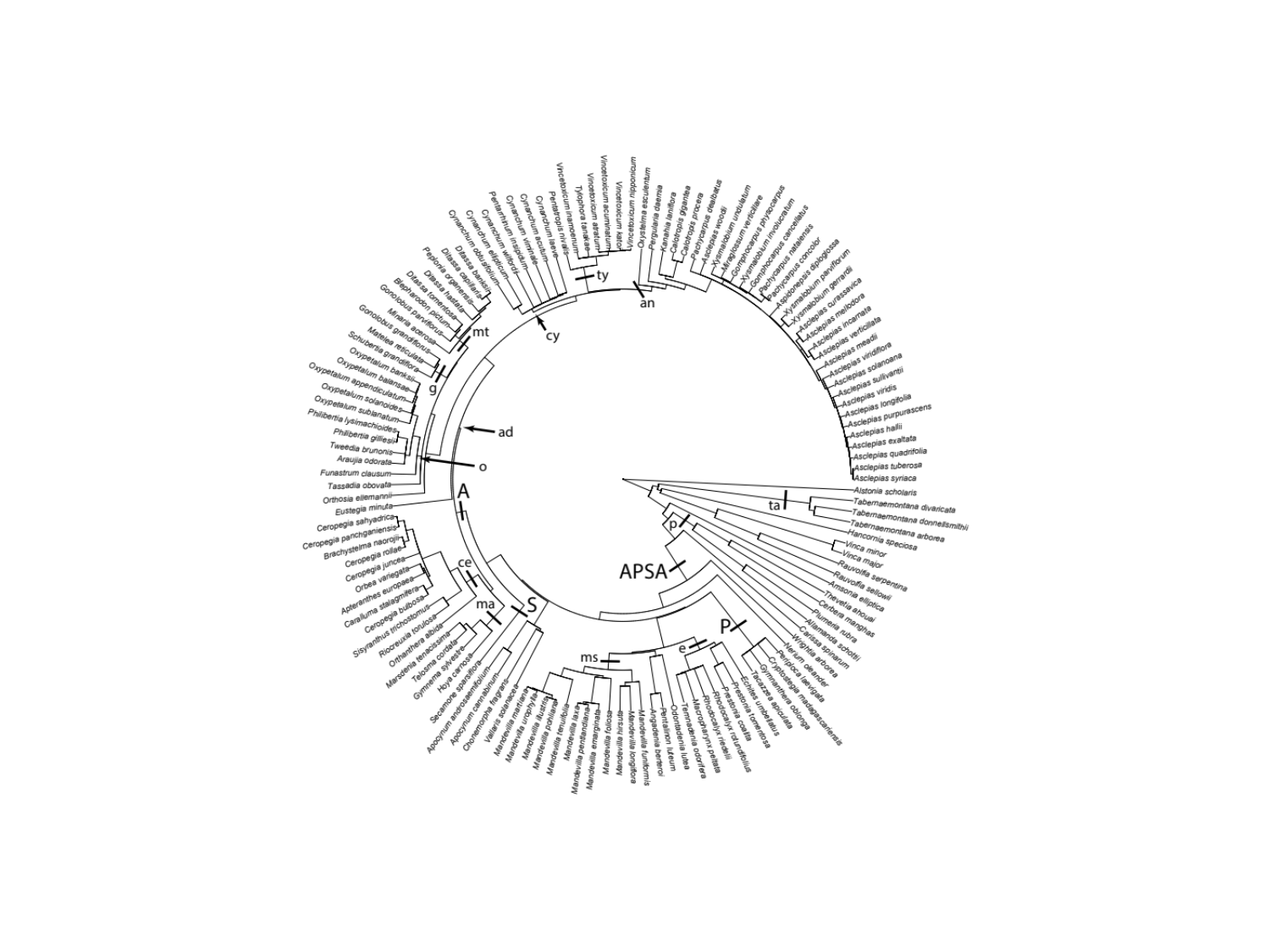

Supplement: Supplementary Material S5 [file mcy127_suppl_supplementary_material-s05.pptx]

## Slide 1
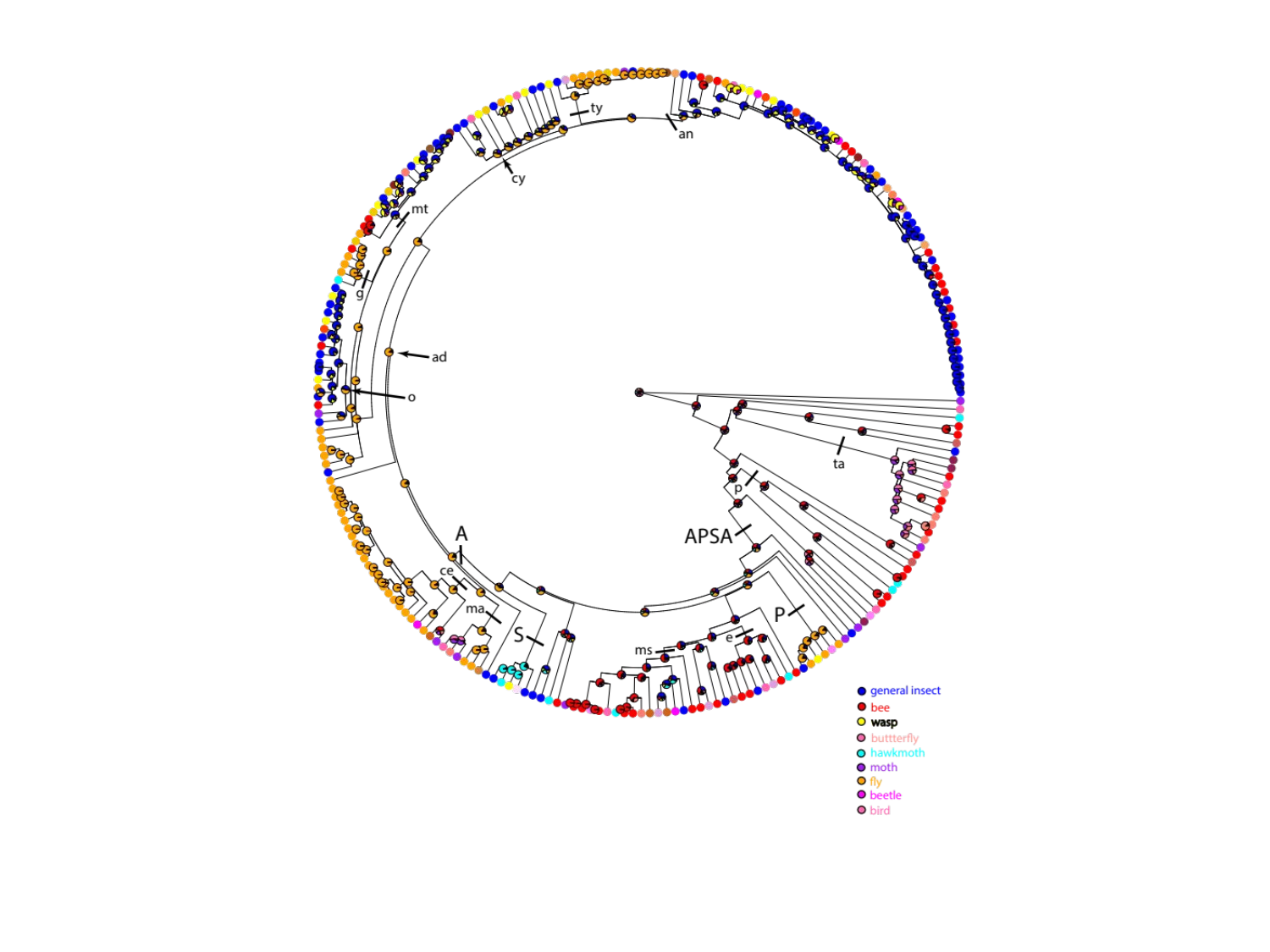

Supplement: Supplementary Material S6 [file mcy127_suppl_supplementary_material-s06.pptx]
